# How Many Separable Sources? Model Selection In Independent Components Analysis

**DOI:** 10.1371/journal.pone.0118877

**Published:** 2015-03-26

**Authors:** Roger P. Woods, Lars Kai Hansen, Stephen Strother

**Affiliations:** 1 Departments of Neurology and of Psychiatry and Biobehavioral Sciences, David Geffen School of Medicine at the University of California Los Angeles, Los Angeles, California, United States of America; 2 Department of Applied Mathematics and Computer Science, Technical University of Denmark, Lyngby, Denmark; 3 Rotman Research Institute, Baycrest, and Department of Medical Biophysics, University of Toronto, Toronto, Canada; Brown University, UNITED STATES

## Abstract

Unlike mixtures consisting solely of non-Gaussian sources, mixtures including two or more Gaussian components cannot be separated using standard independent components analysis methods that are based on higher order statistics and independent observations. The mixed Independent Components Analysis/Principal Components Analysis (mixed ICA/PCA) model described here accommodates one or more Gaussian components in the independent components analysis model and uses principal components analysis to characterize contributions from this inseparable Gaussian subspace. Information theory can then be used to select from among potential model categories with differing numbers of Gaussian components. Based on simulation studies, the assumptions and approximations underlying the Akaike Information Criterion do not hold in this setting, even with a very large number of observations. Cross-validation is a suitable, though computationally intensive alternative for model selection. Application of the algorithm is illustrated using Fisher's iris data set and Howells' craniometric data set. Mixed ICA/PCA is of potential interest in any field of scientific investigation where the authenticity of blindly separated non-Gaussian sources might otherwise be questionable. Failure of the Akaike Information Criterion in model selection also has relevance in traditional independent components analysis where all sources are assumed non-Gaussian.

## Introduction

**Fig 1:**
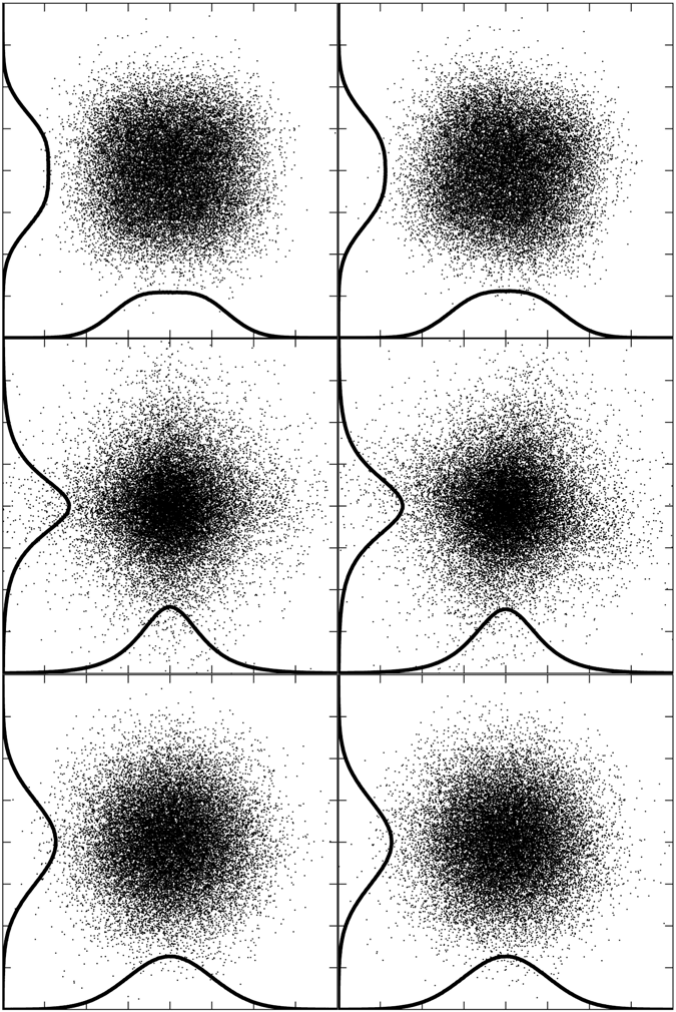
Mixtures of non-Gaussian and of Gaussian sources. Points with x- and y- coordinates drawn from two unmixed source distributions are plotted in the left panel. At the top, both sources were randomly drawn from a sub-Gaussian distribution; in the middle, both from a super-Gaussian distribution; and at the bottom, both from a Gaussian distribution. Corresponding images on the right show the distributions after the sources on the left were remixed with an orthonormal matrix representing a thirty degree rotation. For the non-Gaussian sources, the lack of statistical independence resulting from the rotation can be identified visually even without seeing the unrotated images as a result of the higher order distributional statistics. Statistical independence in the unmixed images is evident from the fact that the two-dimension distribution can be predicted as the product of the illustrated marginal distributions along each individual axis. When both sources are Gaussian, the two-dimensional distribution can be predicted from the marginal distributions of any arbitrary orthogonal pair of coordinate axes, so statistical independence cannot be used as a criterion for identifying the original Gaussian sources.

Independent components analysis (ICA) has recently emerged as a valuable tool for the analysis of multivariate data sets and is increasingly used in a broad array of scientific contexts [1], [2], [3]. ICA techniques utilizing higher order statistics can separate mixtures of sub-Gaussian and/or super-Gaussian signals into their source components, thereby achieving blind source separation. When each individual multivariate observation represents an independent sample, an important limitation of ICA techniques is that Gaussian components, lacking the requisite higher order statistical properties, cannot be separated from one another, as illustrated in Fig. 1. Although it has been argued that true Gaussian sources are uncommon [4], finite distributions that are subtly non-Gaussian may nonetheless behave as if they were Gaussian from the standpoint of blind source separation, particularly for sample sizes typically available for high-dimensional data sets encountered in neuroimaging or gene expression microarrays. If multiple Gaussian components are present in the data but not included in the ICA model, the resulting ICA decomposition of the Gaussian subspace into “sources” will be dictated by random statistical fluctuations. As a result, sources identified by these methods are potentially tainted by concerns that their identification as a non-Gaussian source is not statistically justifiable. The work described here incorporates a potential Gaussian subspace into the ICA model and addresses the problem of model selection in this mixed ICA/PCA framework. Application of this technique to two publicly available multivariate data sets is illustrated. Software implementing these novel features is freely available.

## Methods

### Maximum likelihood ICA formulation

The maximum likelihood formulation of ICA is formally equivalent to ICA based on information maximization, mutual information or negentropy [4]. In this approach, the Kullback-Leibler (K-L) divergence [5] between the model and the observed data is minimized, where K-L divergence is a measure of the dissimilarity between statistical populations [6]. The maximum likelihood framework allows multiple Gaussian components to be formalized as part of the ICA procedure. It will be assumed here that *n* independent multivariate samples (e.g., as might derive from *n* subjects drawn randomly from a population), each consisting of *m* observations is stored in an *m* by *n* observation matrix **X** that is to be subjected to ICA. Subtracting the mean of each row from the elements of that row will yield the matrix $\tilde{X}$ such that the covariance of the original observation matrix is proportional to ${\tilde{X}}^{\text{T}}\tilde{X}$. Additionally, it will be assumed that the rows of $\tilde{X}$ are linearly independent (i.e., that no row of $\tilde{X}$ can be expressed as a linear combination of other rows of $\tilde{X}$), a condition that can be assured through preprocessing with singular value decomposition since matrices related to one another through left multiplication by an orthonormal matrix have identical ICA sources. Such preprocessing also assures that *m*≤*n*. ICA can be described as the decomposition of $\tilde{X}$ into the matrix product A*S, where **A** is an *m* by *m* mixing matrix and **S** is an *m* by *n* source matrix with rows that are maximally independent, but not necessarily orthogonal. The preprocessing already described assures that **A** can be inverted to generate an unmixing matrix **W**. The ICA problem can therefore be restated as the problem of finding **W** such that the sources computed as the matrix product W*X are maximally independent.

The logarithm of the likelihood of **W**’s contribution from the *j*th column of $\tilde{X}$ is [7], [8]:

$$\log\text{P}({\tilde{X}}_{:j})=\log\left|\det W\right|+\sum_{i=1}^{m}\log{\text{p}}_{i}(S_{ij})$$ (1)

where the use of ‘:’ as an index signifies the entire row or column, |det **W**| signifies the absolute value of the determinant of **W**, and p_*i*_(**S**_*ij*_) is the probability of the element of **S** in the *i*th row and *j*th column based on the assumption that the rows of **S** are independent and sampled from a pre-specified non-Gaussian distribution. For a series of *n* such observations, the log likelihood of the entire observed series can therefore be computed as:

$$\log\text{P}(\tilde{X})=n\log\left|\det W\right|+\sum_{j=1}^{n}\sum_{i=1}^{m}\log{\text{p}}_{i}(S_{ij})$$ (2)

To maximize the log likelihood by adjusting the elements of **W**, partial derivatives with respect to these elements are needed. The partial derivative of the log likelihood of the *j*th column of $\tilde{X}$ with respect to **W**_*ik*_ can be computed [8] as:

$$\frac{\partial }{\partial W_{ik}}\log\text{P}({\tilde{X}}_{:j})=A_{ki}+\frac{d\log{\text{p}}_{i}(S_{ij})}{dS_{ij}}{\tilde{X}}_{kj}$$ (3)

It follows that the partial derivative of the log likelihood of the entire observation matrix $\tilde{X}$ with respect to **W**_*ik*_ is given by

$$\frac{\partial }{\partial W_{ik}}\log\text{P}(\tilde{X})=nA_{ki}+\sum_{j=1}^{n}\frac{d\log{\text{p}}_{i}(S_{ij})}{dS_{ij}}{\tilde{X}}_{kj}=nA_{ik}^{\text{T}}+\sum_{j=1}^{n}\frac{d\log{\text{p}}_{i}(S_{ij})}{dS_{ij}}{\tilde{X}}_{jk}^{\text{T}}$$ (4)

If the *m* by *m* matrix **dW** is defined as a matrix that has $\frac{\partial }{\partial W_{ik}}\log\text{P}(\tilde{X})$ in the *i*th row and *k*th column and if the *m* by *n* matrix **dS** is defined as a matrix that has $\frac{d\log{\text{p}}_{i}(S_{ij})}{dS_{ij}}$ in the *i*th row and *j*th column, then

$$dW=nA^{\text{T}}+dS*{\tilde{X}}^{\text{T}}=n{\left(W^{\text{T}}\right)}^{-1}+dS*{\tilde{X}}^{\text{T}}$$ (5)

When the log likelihood is maximized, **dW** becomes a matrix of zeros. Operationally, this means that ICA decomposition can be implemented using a gradient descent algorithm to minimize the negative log likelihood of the observed values of $\tilde{X}$ by modifying the elements of the matrix **W** using the derivatives of the negative log likelihood with respect to those elements. When the log likelihood is maximized, the sources will satisfy:

$$n*I=-dS*{\tilde{X}}^{\text{T}}*W^{\text{T}}=-dS*S^{\text{T}}$$ (6)

where **I** is an *m* by *m* identity matrix. Moreover, for a given row of **dW** to consist entirely of zeros, it is sufficient that -**dS**_*i*:_ * **S** ^T^ = n*I_*i*:_ since

$$dW=nA^{T}+dS*{\tilde{X}}^{T}=nA^{T}+dS*S^{T}*A^{T}$$ (7)

A practical consideration that is of particular concern when modeling more than one type of source distribution is that a gradient descent algorithm may converge to a local optimum that is not the global optimum. This is a known problem with ICA even without the inclusion of Gaussian components [9]. The primary approach to this problem that will be utilized here is to initialize the optimization for a given number of sub-Gaussian, super-Gaussian and Gaussian sources multiple times, selecting the final result for that combination of sources from the optimization producing the largest log likelihood. Conceptually, the various initializations are selected by reordering the rows of $\tilde{X}$ such that the matrix **W** (specified as an identity matrix at the start of each individual initialization) maps the rows of $\tilde{X}$ to every possible combination of source labels. In practice, this is achieved with greater computational efficiency by maintaining the original order of the rows of $\tilde{X}$ and instead exchanging the columns of the identity matrix used to initialize **W**. In some instances, this primary approach is insufficient, and a secondary approach is required. For the secondary approach, a random *m* by *m* orthonormal matrix **O**_*R*_ is generated, and the ICA sources of the matrix product $O_{R}*\tilde{X}$ are computed as (W*OR')*(OR*X˜). When using this secondary approach, which can be repeated for any desired number of random matrices **O**_*R*_, the primary approach described above can still be used when computationally feasible to initialize the gradient descent optimization of the elements of **W** * **O**_*R*_'.

### Source distributions

Central to ICA methods based on higher order statistics is the observation that the particular probability distribution used to model non-Gaussian sources has little impact on the resulting decomposition, making one heavy tailed super-Gaussian distribution and one light tailed sub-Gaussian distribution sufficient to separate mixtures of super-Gaussian and sub-Gaussian sources [10], [11]. Here a Gaussian source distribution is also included, with all three distributions adjusted to have identical variance. Specifically, the super-Gaussian distribution $\text{p}(x)=\frac{1}{2\text{cosh}(\frac{\pi x}{2})}$, the sub-Gaussian distribution $\text{p}(x)=\frac{1}{\sqrt{\pi e}}e^{-x^{2}}\cosh(x\sqrt{2})$, and the Gaussian distribution $p(x)=\frac{1}{\sqrt{2\pi }}e^{\frac{-x^{2}}{2}}$ are modeled. For these three distributions, elements of the matrix **dS** can be computed as $dS_{ij}=-\frac{\pi }{2}\tanh(S_{ij}\frac{\pi }{2})$, $dS_{ij}=-2S_{ij}+\sqrt{2}\tanh(S_{ij}\sqrt{2})$, and **dS**_*ij*_ = -**S**_*ij*_, respectively. The problem of identifying *m*_*1*_ super-Gaussian sources, *m*_*2*_ sub-Gaussian sources and *m*_*3*_ Gaussian components therefore requires finding the unmixing matrix **W** that maximizes:

$$\begin{array}{l} \mathrm{logP}(\tilde{X})=n\log\left|\det W\right|\\ -nm_{1}\log(2)-\sum_{j=1}^{n}\sum_{i=1}^{m_{1}}\log(\cosh(S_{ij}\frac{\pi }{2}))\\ -\frac{nm_{2}}{2}\left(\log(\pi )+1\right)+\sum_{j=1}^{n}\sum_{i=m_{1}+1}^{m_{2}}\left(-S_{ij}^{2}+\log(\cosh(S_{ij}\sqrt{2}))\right)\\ -\frac{nm_{3}}{2}\left(\log(2\pi )\right)-\frac{1}{2}\sum_{j=1}^{n}\sum_{i=m_{1}+m_{2}+1}^{m}S_{ij}^{2} \end{array}$$ (8)

### Properties of the Gaussian Subspace

As noted above, **dS**_*ij*_ = -**S**_*ij*_ for the Gaussian sources. Combined with the requirement that-**dS** * **S** ^T^ = *n* * **I** when the log likelihood is maximized, this leads to the conclusion that maximization of the log likelihood requires every Gaussian component to be orthogonal to all other sources (including all non-Gaussian sources). It also requires that the norm of each Gaussian component be equal to $\sqrt{n}$. From this, it follows that $\sum_{j=1}^{n}\sum_{i=m_{1}+m_{2}+1}^{m}S_{ij}^{2}=nm_{3}$, so the ICA optimization problem is reduced to finding **W** that maximizes:

$$\begin{array}{l} \mathrm{logP}(\tilde{X})=n\log\left|\det W\right|\\ -nm_{1}\log(2)-\sum_{j=1}^{n}\sum_{i=1}^{m_{1}}\log(\cosh(S_{ij}\frac{\pi }{2}))\\ -\frac{nm_{2}}{2}\left(\log(\pi )+1\right)+\sum_{j=1}^{n}\sum_{i=m_{1}+1}^{m_{2}}\left(-S_{ij}^{2}+\log(\cosh(S_{ij}\sqrt{2}))\right)\\ -\frac{nm_{3}}{2}\left(\log(2\pi )+1\right) \end{array}$$ (9)

When two or more Gaussian sources are modeled, the unmixing matrix **W** is not unique; linear recombination of the Gaussian components using any orthonormal rotation matrix **R** that does not alter the non-Gaussian sources will produce the same value for $\log P\left(\tilde{X}\right)$ since the $\sum_{j=1}^{n}\sum_{i=m_{1}+m_{2}+1}^{m}S_{ij}^{2}$ term in Equation 8 will be unchanged if **S** is replaced by R*S and the det **W** term will likewise be unchanged if **W** is replaced by W*R ^T^. The rotational invariance of $\sum_{j=1}^{n}\sum_{i=m_{1}+m_{2}+1}^{m}S_{ij}^{2}$ is the reason that higher order statistics cannot separate multiple Gaussian components from one another using ICA.

This rotational invariance of the Gaussian contributions also allows the Gaussian components to be linearly recombined using PCA criteria. If the last *m*_*3*_ sources in **S** correspond to a Gaussian distribution, the last *m*_*3*_ columns of **A** account for the contribution of these sources to $\tilde{X}$. These columns of **A** can be decomposed using singular value decomposition into the product U*D*V ^T^ where **V** is an orthonormal *m*_*3*_ by *m*_*3*_ matrix. If **R** is defined by replacing the last *m*_*3*_ rows of the last *m*_*3*_ columns of an *m* by *m* identity matrix with **V** ^T^, the mixed ICA/PCA decomposition **X = A**_ICA/PCA_*S_ICA/PCA_ can be computed by setting **A**_ICA/PCA_ = A*R ^T^ and **S**_ICA/PCA_ = R*S. The last *m*_*3*_ rows of **S**_ICA/PCA_ will account for progressively smaller amounts of variance in $\tilde{X}$, and will be eigenvectors of ${\tilde{X}}^{\text{T}}\tilde{X}$. In what follows, the ICA/PCA subscript will be omitted from **A** and **S** and the term “Gaussian component” should be understood to refer to an eigenvector of ${\tilde{X}}^{\text{T}}\tilde{X}$ and not to imply that Gaussian sources have been separated when multiple Gaussian components are modeled.

When Gaussian components are included in the model, it is not necessary to rely on the optimization algorithm to iteratively converge on a solution such that the Gaussian components are orthogonal to all other sources. Instead, the optimization algorithm can be used to modify only the elements of **W** that generate the non-Gaussian sources and QR decomposition can be used to solve for the remaining rows subject to the orthogonality constraints and the requirement that $S=W*\tilde{X}$. The orthogonality constraints will assure that -**dS**_*i*:_ *S ^T^ = **S**_*i*:_ *S ^T^ = n*I_*i*:_ for the Gaussian rows of **S**, and it follows that the corresponding rows of **dW** will consist entirely of zeros. Since the derivatives of $\mathrm{logP}(\tilde{X})$ with respect to the rows of **W** that generate the Gaussian components are always zero, these elements of **W** do not need to be included among the parameters that are iteratively optimized by gradient descent. Once the log likelihood has been fully maximized, PCA can be used to find an orthonormal linear recombination of the Gaussian rows of **S** such that each row explains the maximal amount of residual variance associated with the Gaussian subspace.

### Model selection

For any given number of sub-Gaussian, super-Gaussian and Gaussian sources, adjusting the model parameters to maximize the log-likelihood will minimize the K-L divergence between the observed data and the model. However, in choosing among models with differing numbers of each type of source, it is not appropriate to simply select the model with the largest maximized log-likelihood. As estimators of the relative K-L divergence of each model from the truth (i.e., from the best possible model, which is not necessarily among the models being evaluated), the maximized log likelihoods are biased, underestimating the true K-L divergences. The magnitude of this bias varies across models, and models with larger numbers of parameters generally have larger biases. A common approach to this problem is to base model selection on a penalized adjustment to the maximized log-likelihoods, where the penalty term is a function of the number of model parameters. A variety of penalty adjustments have been proposed and differ in terms of their underlying goals, assumptions and approximations.

Before discussing specific model selection strategies, it is important to emphasize several pertinent aspects of the mixed ICA/PCA model selection problem. First of all, it should be noted that the models involving different numbers of sub-Gaussian, super-Gaussian and Gaussian sources are not nested models in the sense that one model is a specialized case of another model. Moreover, as noted in the previous section, when the true model includes two or more Gaussian sources, they occupy a Gaussian subspace that is orthogonal to all other sources, and they cannot be separated using ICA. When incorrectly modeling one or more of the components of this subspace as if they were non-Gaussian, the ICA sources associated with this subspace are the result of random noise, and thus, from a purely theoretical standpoint, are not reproducible. Finally, it should be noted that all of the models under consideration will typically be misspecified, with truth not among the models under consideration since it is unlikely that the specific sub-Gaussian and super-Gaussian distributions implemented in the ICA model will coincide with the actual source distributions. The utility of ICA as a data analysis technique rests on the well-known robustness of the identified sources to misspecification of the source distributions.

One commonly used penalized log-likelihood model selection strategy is the Bayes Information Criterion (BIC) of Schwarz [12], which subtracts the penalty term $K\frac{\log(n)}{2}$ from the maximized log likelihoods, where *K* is the number of parameters in the model. Derived from Bayesian considerations, the BIC is one of a class of log-likelihood penalty terms designed to achieve *consistency* in model selection, where a consistent criterion is defined as one that assures that the probability of selecting the model closest to truth increases to one as *n* increases to infinity. Defined only as an asymptotic limit, consistency *per se* does not provide any assurances regarding the probability of selecting the best model for any finite sample size. For mixed ICA/PCA, the asymptotic limit for suitably chosen non-Gaussian source distributions is arguably already known, since virtually all real-world sources are thought to be non-Gaussian given a sufficiently large sample size [4]. The goal of model selection in mixed ICA/PCA is not to identify those rare instances in which real-world sources are Gaussian, but rather to assure that all observed data dimensions subjected to ICA are sufficiently non-Gaussian to be unlikely to have been generated by Gaussian sources. Even if sources are known for a fact to be non-Gaussian, if they cannot be distinguished from mixtures of Gaussian sources empirically using the observed data, any ICA decomposition of the observed data should be regarded as highly suspect given that any ICA decomposition of a mixture of Gaussian sources is necessarily random and therefore not reproducible.

The goals of model selection in mixed ICA/PCA are better aligned with an alternative set of penalized log-likelihood methods designed to minimize the K-L divergence between the observed data and truth. The Akaike Information Criterion (AIC), which subtracts the number of modeled parameters as a penalty term to adjust the maximized log-likelihoods, is one such model selection method [13]. Unlike the BIC, the AIC is not asymptotically consistent, so it is not well-suited for evaluating whether real-world sources are truly non-Gaussian. Instead, for large but finite sample sizes, the AIC is well-suited for determining whether observed data is more consistent with having originated from mixtures of Gaussian or non-Gaussian sources. Derivation of the AIC involves certain assumptions and approximations, including, but not limited to a large observed sample size and a correctly specified model. Some of these limitations have been addressed through problem domain-specific refinements. For example, as will be discussed later, small sample sizes and misspecified models have been very well addressed by modifications to AIC in the context of multivariate regression. Before considering whether AIC can be similarly modified to address model selection in mixed ICA/PCA, it is relevant to summarize the underlying motivation for the AIC approach.

Following the AIC notational conventions of Burnham & Anderson [6], given a set of *n* multivariate observations *y*, the parameters of a candidate model can be adjusted to find the values $\underline{\hat{\theta }}\left(\underline{y}\right)$ that maximize the log likelihood of *y* under the model. As already noted, the log likelihood associated with these parameters is biased. To eliminate this bias, a new set of *n*_2_ independent observations (typically, but not necessarily with *n*_2_ = *n)*, *x* can be drawn from the same distribution as *y*, and the parameters that were originally optimized using *y* can then be used to calculate the probability *g* of the new observations $g\left(\underline{x}|\hat{\underline{\theta }}\left(\underline{y}\right)\right)$. Taking the logarithm of the result produces an unbiased estimate of the log likelihood of the model,$\log\left[g\left(\underline{x}|\hat{\underline{\theta }}\left(\underline{y}\right)\right)\right]$. This estimate is unbiased in the sense that repeating the process with new sets of independent observations *x*_1_, *x*_2_, … will tend to produce the same log likelihood for a given $\underline{\hat{\theta }}\left(\underline{y}\right)$. Thus there is an expected value for the unbiased log likelihood for the parameters derived from *y*, ${\text{E}}_{\underline{x}}\left[\log\left[g\left(\underline{x}|\hat{\underline{\theta }}\left(\underline{y}\right)\right)\right]\right]$ for which $\log\left[g\left(\underline{x}|\hat{\underline{\theta }}\left(\underline{y}\right)\right)\right]$ obtained from any particular *x* is an unbiased estimator. This quantity, though unbiased, is still only a sample estimate in the sense that it is predicated on the initial selection of *y*. Repeating the entire process with independent sets of *n* observations *y*_1_, *y*_2_, … will tend to produce similar results centered around some expected value ${\text{T}}_{n}\equiv {\text{E}}_{\underline{y}}{\text{E}}_{\underline{x}}\left[\log\left[g\left(\underline{x}|\hat{\underline{\theta }}\left(\underline{y}\right)\right)\right]\right]$. The model with largest estimated value of T_*n*_ is the model that minimizes K-L divergence from truth, and ${\hat{\text{T}}}_{n}=\log\left[g\left(\underline{x}|\hat{\underline{\theta }}\left(\underline{y}\right)\right)\right]$ is an unbiased estimate of T_*n*_. The assertion that this estimate is unbiased ignores any bias associated with the model selection process itself, a problem intrinsic to any data driven model selection procedure [14]. Burnham & Anderson [6] have discussed this issue in the context of the AIC and argue that such bias is small.

The subscript *n* is used in T_*n*_ to emphasize that T is a function of the sample size *n*. From a practical standpoint, this means that if 2*n* observations are collected and half are treated as *y* and half as *x*, the resulting unbiased estimate T_*n*_ will only be relevant for *n* observations, not for the full 2*n* observations that were collected. Since larger numbers of observations will generally tend to produce better support for more complex models, such a strategy effectively wastes half of the data collected and is biased towards simpler models than could be supported by all 2*n* observations. Under certain circumstances (specifically, those assumed in deriving the AIC or domain-specific refinements to the AIC), it is possible to directly estimate the bias of $\log\left[g\left(\underline{y}\right)|\hat{\underline{\theta }}\left(\underline{y}\right)\right]$ as an estimator of T_*n*_ without requiring a second independent set of observations. When the bias can be estimated with reasonable accuracy, the optimized log likelihood of all observations can be corrected to generate an approximately unbiased estimate of T_*n*_ suitable for model selection. To evaluate the accuracy with which the bias of T_*n*_ might be estimated from a single set of observations in the context of ICA/PCA, it is informative at this point to consider simulated data in which the true source distributions are known.

**Fig 2:**
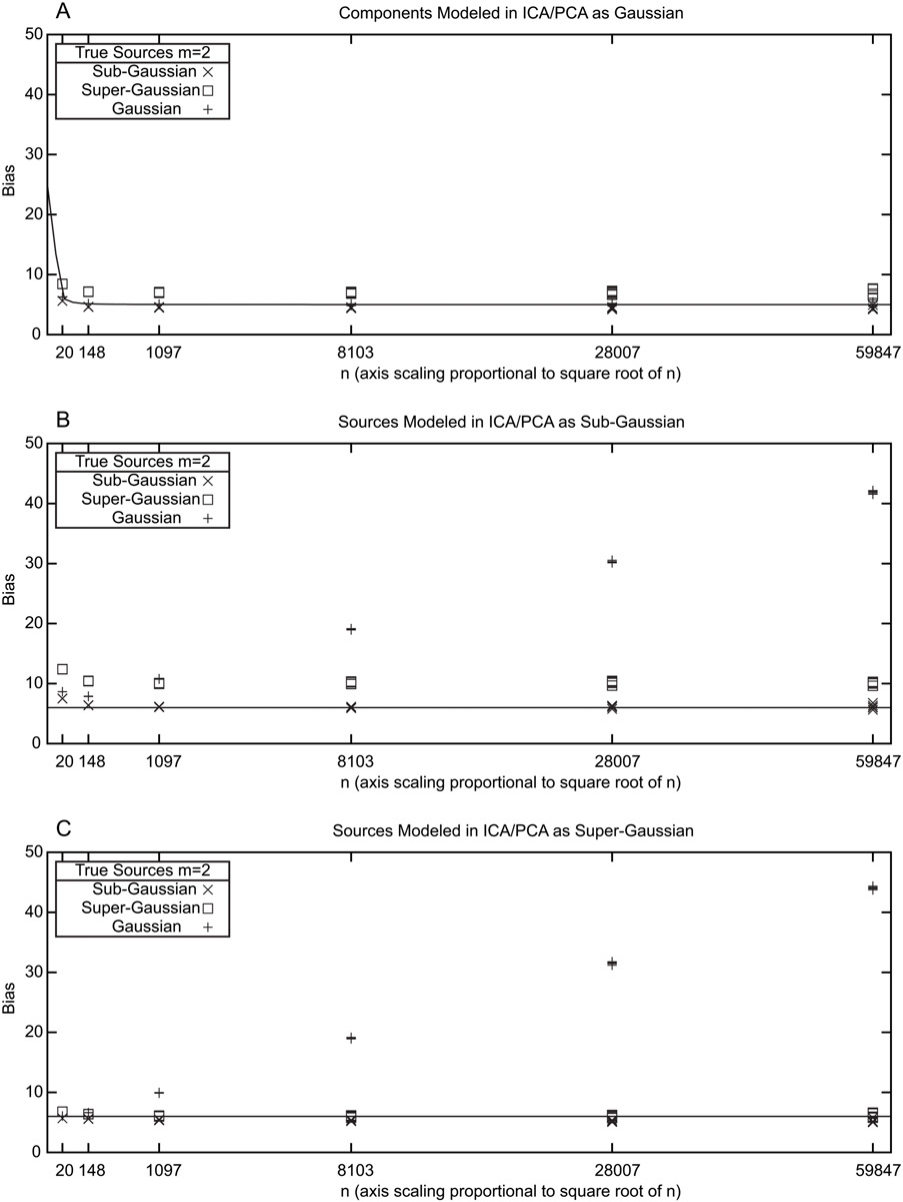
Estimation of ${\hat{\text{T}}}_{n}$ bias in simulated data. Each plotted data point represents one million simulations and five such points are plotted for each model. In each simulation, *m* was two and the value of *n* is shown on the x-axis. Sampling along the x-axis is logarithmic but the scaling is proportional to $\sqrt{n}$. The biases were calculated by first solving for **W** using a set of *n* random deviates from the designated true source distribution and comparing the resulting log likelihood with the log likelihood estimated from a new independent set of *n* random deviates from the same distribution using the value of **W** derived from the first set. **A.** Results with both sources modeled as Gaussian. The solid line shows predicted results based on Fujikoshi and Satoh (1997). **B.** Results with both sources modeled as sub-Gaussian. **C.** Results with both sources modeled as super-Gaussian. The solid lines in B and C reflect the asymptotic Akaike prediction of a bias of 6.

Fig. 2A shows results of simulated data for *m* = 2 and various values of *n* using an ICA/PCA model that consists exclusively of Gaussian components (i.e., effectively just PCA). For each of the *m* = 2 rows, three different source distributions were used in independent simulations, one Gaussian, one sub-Gaussian and one super-Gaussian. Gaussian random deviates were generated using the Mersenne twister pseudorandom number generator of Matsumoto and Nishimura [15]. This pseudorandom generator generates uniformly distributed random numbers on the closed interval [1] and these were used in turn to generate normally distributed random numbers with a mean of zero and unit variance using the polar form of the Box-Muller transformation [16]. Pseudo-random deviates from the sub-Gaussian distribution $p(x)=\frac{1}{\sqrt{\pi e}}e^{-x^{2}}\cosh(x\sqrt{2})$ were generated by randomly adding or subtracting $\frac{1}{\sqrt{2}}$ to Gaussian random deviates with a mean of zero and variance of $\frac{1}{2}$. Pseudo-random deviates from the super-Gaussian distribution $p(x)=\frac{1}{2cosh(\frac{\pi x}{2})}$, were generated by converting uniform random deviates *u* in the open interval (0,1) generated using the Mersenne twister pseudorandom generator to super-Gaussian deviates *v* using the transformation $v=\frac{4}{\pi }*\tanh^{-1}\left(\tan\left(\frac{\pi }{2}\left(u-\frac{1}{2}\right)\right)\right)$. Note that the sub-Gaussian and super-Gaussian deviates were deliberately generated from the same distributions that are used in the ICA/PCA model, an unrealistic best-case scenario from the standpoint of model misspecification. For each value of *n* and each type of source distribution, five independent sets of simulations were performed to estimate the bias in T_*n*_, each based on one million independent estimates. For each estimate, two sets of *m* by *n* simulated observations were prepared. For simplicity, an identity matrix was used as **A** to mix the simulated sources in each case. The first set of simulated observations was used to estimate the parameters of the model (specifically, the mean value of each row and the elements of the unmixing matrix **W**) and the biased log likelihood. The second set of observations was used to estimate the unbiased log likelihood. Subtracting the biased and unbiased estimates produced an unbiased estimate of the bias. For *m* = 2 and a pure Gaussian ICA/PCA model, the total number of freely adjustable parameters is five, two associated with centering each row of observations and three associated with the four elements of **W** (orthogonality constraints for Gaussian components makes one element of **W** fully dependent on the other three when all sources are modeled as Gaussian).

The results in Fig. 2A provide a basis for initiating discussion of methods for estimating the bias of $\log\left[g\left(\underline{y}\right)|\hat{\underline{\theta }}\left(\underline{y}\right)\right]$ as an estimator of T_*n*_. First, when the true distribution is Gaussian (therefore matching the modeled distribution), as *n* grows large, the bias asymptotically approaches a value of five. This conforms exactly to work by Akaike [13] indicating that the bias should asymptotically approach the number of modeled parameters. This prediction is the basis for use of the Akaike Information Criterion (AIC), which adjusts the log-likelihood downward by subtracting the number of modeled parameters in model selection. However, as *n* decreases, the bias grows larger than the number of modeled parameters, a well-recognized shortcoming of the AIC. This shortcoming has been addressed in the specific context of multivariate regression with normal residuals by Fujikoshi and Satoh [17] who derived an exact expression for the bias as a function of *n*. Conveniently, when all sources in the ICA/PCA model are Gaussian (i.e., when no non-Gaussian sources are present), the expression for the log likelihood reduces to a formula that is formally equivalent to the maximum log likelihood solution **C** of the multivariate least squares regression problem:

$$X=C*\left[\begin{array}{ccc} 1 & \ldots & 1 \end{array}\right]$$ (10)

where the matrix on the right is a 1 by *n* matrix of ones and **X** is the same matrix **X** subjected to ICA/PCA prior to centering. The solution **C** is an *m* x 1 matrix such that the value in each row of **C** is the mean of the corresponding row of **X**. In this case, the bias can be computed exactly using the result from Fujikoshi and Satoh as:

$$\frac{n\left(m+\frac{m\left(m+1\right)}{2}\right)}{n-m-2}.$$

As *n* increases to infinity, this value converges asymptotically to $m+\frac{m(m+1)}{2}$. For any value of *m*, the first asymptotic term, *m* corresponds to the number of centering parameters, and the second term, $\frac{m\left(m+1\right)}{2}$ corresponds to the number of freely adjustable parameters in **W** after accounting for orthogonality constraints. Consequently, asymptotic convergence is always to the total number of freely adjustable parameters. The solid line in Fig. 2A shows the exact bias calculated using this equation.

Fig. 2A also illustrates that when the true distribution is not Gaussian, Akaike’s asymptotic result is only approximate. From the formal equivalence with multivariate regression, it is known that the asymptotic bias in this case differs from the number of freely adjustable model parameters by $\frac{\kappa_{4}}{2}$, where *κ*_4_ is the multivariate kurtosis of the true distribution [18]. For *m* = 2, the multivariate kurtosis of the sub-Gaussian distribution is -1, leading to an asymptotic bias of 4.5 and the multivariate kurtosis of the super-Gaussian distribution is 4, leading to an asymptotic bias of 7. The multivariate kurtosis of a multivariate Gaussian distribution is always zero.

Unfortunately, when non-Gaussian sources are included in the ICA/PCA model, the bias of ${\hat{\text{T}}}_{n}$ is more problematic. Fig. 2B and Fig. 2C show results comparable to those in Fig. 2A except that the sources were both modeled as sub-Gaussian (Fig. 2B) or as super-Gaussian (Fig. 2C). In these cases, a total of six freely adjustable parameters are modeled (two associated with centering the two rows of **X** and four associated with the elements of **W**). When the modeled distribution matches the true distribution, Akaike’s asymptotic result holds, with biases converging to a value of six. Biases are larger for small values of *n*. When super-Gaussian sources are modeled as sub-Gaussian or vice versa, the asymptotic bias differs from the number of freely adjustable parameters; theoretical results are not currently available to compute the exact asymptotic bias. Most strikingly, when Gaussian components are modeled as sub-Gaussian or super-Gaussian, the bias of T_*n*_ does not appear to asymptotically converge to a finite value at all, but instead empirically involves an additional term that increases in proportion to $\sqrt{n}$ over a broad range of *n*’s for both types of non-Gaussian models. Simulations with *m* = 4 and *m* = 8 (not shown) similarly demonstrate bias increases proportional to $\sqrt{n}$ when Gaussian components were modeled as sub-Gaussian or super-Gaussian. Since the presence or absence of this $\sqrt{n}$ term depends on the same unknown that the unbiased log likelihood is being used to address (namely whether or not the true sources are Gaussian), even an exact analytical expression for the slope of this term would not suffice to unambiguously estimate T_*n*_. Consequently, an AIC-like bias correction in which the log-likelihood is corrected by some additive factor is not suitable for model selection in mixed ICA/PCA.

An alternative approach to estimating T_*n*_ is the cross-validation approach first proposed by Stone [19]. The essential feature of this approach is recognition of the fact although it is not always possible to compute an unbiased estimate that is applicable to *n* samples, ${\hat{\text{T}}}_{n}$, it is always possible to compute an unbiased estimate applicable to *n*-1 samples,${\hat{\text{T}}}_{n-1}$. By holding back one sample when optimizing the model parameters, this held back sample can subsequently be used to generate an unbiased estimate of $\frac{{\text{T}}_{n-1}}{\text{n-1}}$. Since *n* different observations can be left out, this procedure can be repeated for a total of *n* estimates. Adding these *n* estimates gives $\frac{n}{n-1}{\hat{\text{T}}}_{n-1}$, which cross-validation uses as an estimate of T_*n*_. Although this estimate is not unbiased, the magnitude of the bias is generally small. Using the subscript [-*j*] to denote a parameter estimated by omitting the *j*th observation and [+*j*] to denote values derived from the held back *j*th observation **X**_:j_ using parameters estimated without it (centering parameters and **W)**, the estimate is given by:

$$\begin{array}{l} {\text{T}}_{n}\approx \frac{n}{n-1}{\hat{\text{T}}}_{n-1}=\sum_{j=1}^{n}\log\left|\det W_{\left[-j\right]}\right|\\ -nm_{1}\log(2)-\sum_{j=1}^{n}\sum_{i=1}^{m_{1}}\log(\cosh(S_{i\left[+j\right]}\frac{\pi }{2}))\\ -\frac{nm_{2}}{2}\left(\log(\pi )+1\right)+\sum_{j=1}^{n}\sum_{i=m_{1}+1}^{m_{2}}\left(-S_{i\left[+j\right]}^{2}+\log(\cosh(S_{i\left[+j\right]}\sqrt{2}))\right)\\ -\frac{nm_{3}}{2}\left(\log(2\pi )\right)-\frac{1}{2}\sum_{j=1}^{n}\sum_{i=m_{1}+m_{2}+1}^{m}S_{i\left[+j\right]}^{2} \end{array}$$ (11)

The advantage of the cross-validation approach is that it does not require knowledge or assumptions about the true nature of the source distributions making it robust in the presence of misspecification; this advantage comes at the expense of an approximately *n*-fold increase in computations. When multiple initializations of the algorithm are being used to avoid local optima, this *n*-fold increase can be partially offset by assuming that local optima sufficiently close together are identical and only estimating the bias once for a collection of virtually identical local optima; pragmatically, this can be accomplished with little additional computational expense by identifying solutions that have the same uncorrected log likelihood and the same determinant of the unmixing matrix **W** to some arbitrary number of decimal places. Since cross validation utilizes an unbiased estimate of T_*n*-1_, the empirically observed bias term proportional to $\sqrt{n}$ when Gaussian sources are modeled as non-Gaussian in ICA/PCA does not enter into the analysis, having already been taken into account by the estimation procedure.

Since cross-validation provides an AIC-like correction factor, the tradition in the model selection literature would favor doubling the bias adjusted log-likelihood of each model and defining this as an information criterion. This is in conflict with the tradition in the ICA literature, where bias is typically ignored (which would be valid if bias did not vary among the models being compared) and the focus is on minimizing K-L divergence, which does not involve doubling the log-likelihood. The approach taken here is to remain consistent with the ICA literature by not doubling the bias-corrected log-likelihoods. As a result, differences in log-likelihoods should be interpreted keeping in mind that they correspond to information criterion differences that are twice as large. Burnham and Anderson [6] suggest that AIC differences of 0–2 provide substantial empirical support for the alternative model, that AIC differences of 4–7 provide considerably less empirical support and that AIC differences greater than 10 provide essentially no empirical support. Eliminating the gaps between categories, the approach here for the examples will be to consider alternative models with log-likelihood differences less than 2 compared to the best fitting model to be reasonable alternatives that should be seriously considered, those with log-likelihood differences from 2–5 to be plausible alternatives and those with log-likelihood differences greater than 5 to be unlikely alternatives.

### High Dimensional Data

The mixed ICA/PCA approach outlined above is computationally demanding at several levels. While most of the demands, including those associated with computation of bias using cross-validation, increase linearly with the number of subjects, *n*, demands increase far more rapidly with the number of observations per subject, *m*. The number of model categories to consider grows as m*(*m*+1)/2, and within a model category, the maximum number of parameters in the unmixing matrix to be optimized increased as *m* ^2^. Most restrictive is the fact that the number of initializations associated with the primary strategy for avoiding local minima grows as (*m*_1_+*m*_2_+*m*_3_)!/ (*m*_1_!*m_2_!*m_3_!), which practically limits *m* to values of fifteen or fewer to keep the number of initializations below one million. It is important to emphasize that the validity of the log likelihood and bias estimation procedures are in no way dependent on use of this primary strategy—random orthonormal rotations (the secondary strategy) with any desired number of rotations can be substituted with the caveat that the chances of failing to find the globally best solution decrease as the number of initializations decreases. No matter how few random orthonormal rotations are tried, the best fitting bias corrected model will represent the best known model, and the difference in bias corrected log likelihoods relative to the best known model will provide an appropriate estimate of its relative plausibility.

A variety of other strategies can be used to reduce computational demands. When *m* is large, singular value decomposition can be used to reduce *m* to a more tractable value by eliminating a multidimensional orthogonal subspace containing the smallest possible amount of variance before performing mixed ICA/PCA, a strategy also commonly employed in standard ICA. This approach effectively ignores any non-Gaussian signals within the excluded subspace. Since the mixed ICA/PCA algorithm alone is already able to identify a Gaussian subspace when justified by model selection criteria, dimension reduction prior to mixed ICA/PCA is undesirable and should be avoided when possible if the goal is to maximize the chances of identifying all non-Gaussian sources in the original data. Nonetheless, multidimensional data that has been reduced in dimensionality using singular value decomposition is valid data, and any non-Gaussian sources in the best fitting bias corrected model identified after dimensionality reduction can be considered an approximation of sources in the original data. Since dimensionality reduction simply imposes an added constraint, any model derived from the reduced data has an exact corresponding model for the complete original data set, so once the excluded Gaussian subspace is taken into account, the original data set will always have an equally good model and will typically have a better model than any model obtained from the reduced data.

Avoiding the need to estimate bias using cross-validation is another potential strategy for reducing computation. The optimal bias corrected log likelihoods for very similar model categories (i.e., categories that differ by shifting just one source or component between Gaussian, sub-Gaussian or super-Gaussian designatinos) are likely to be similar to one another, so the range of possible categories can be sparsely sampled initially and refined through denser sampling in the vicinity of the best sparse results. Also, given that the AIC generally underestimates the actual bias, initializations for which the AIC corrected log likelihood already suggests an implausible result relative to the best known cross-validation corrected result can be discarded without performing the cross-validation step.

Once a good model is identified, some or all of the non-Gaussian sources from that model can be used to initialize the search for better nearby models by initializing the appropriate elements of the unmixing matrix with the values needed to generate those sources while the remaining elements are initialized with an orthonormal basis that spans the remaining dimensions. Since all elements of the unmixing matrix remain subject to optimization, the non-Gaussian sources used for initialization can be updated by the optimization procedure. Initialization of the orthonormal basis for other sources can be varied using the same strategies already described for unconstrained initializations. This strategy is particularly well-suited for looking to improved local minima by searching for nearby models in the same or similar distributional categories.

Another potential strategy in the cross-validation phase is rather than to perform the leave-one-out strategy by leaving out all *n* samples in turn, to instead only choose some random subset of *n* for the leave-one-out cross-validation procedure and to then rescale the results accordingly on the assumption that the subset is representative of the whole sample. Unlike k-fold cross-validation, where multiple samples are left out simultaneously, this strategy assures that the sample size used for the bias estimation still differs only be one from the full sample size, thereby conforming as closely as possible to ICA’s stated goal of optimizing K-L divergence. However, the random subset may need to be quite large in order to accurately represent the full sample in the ICA context.

### Implementation

The mathematical concepts described here for mixed ICA/PCA have been implemented in C and made accessible in R as an extension package ‘icapca’ available through the Comprehensive R Archive Network (http://www.r-project.org/). A quasi-Newton unconstrained linear optimization algorithm [20] is used to maximize the likelihood function. While the default is to implement the entire mixed ICA/PCA procedure to run serially on a single processor, the package includes options that can be used to break the problem down into smaller components that can be run in parallel. This can be done either at the granularity of a single model category (i.e., a specified number of sub-Gaussian, super-Gaussian and Gaussian components) or at the smaller granularity of a single initialization within a single model category.

## Results

### Example 1: Iris Sepal and Petal Measurements

**Fig 3:**
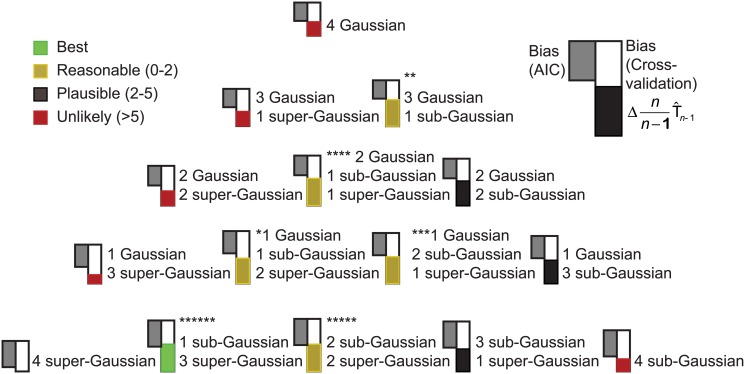
Cross-validation results for the iris dataset. For each of the fifteen model categories, the best fitting model within the category is shown. The solid bar shown in color on the right represents the increase in $\frac{n}{n-1}{\hat{\text{T}}}_{n-1}$ relative to the poorest fitting category, and the solid white bar above it represents the estimated bias in the optimized log-likelihood of the model as an estimate of T_*n*_. The bar on the left shows the AIC bias estimate based on the number of freely adjustable parameters in the model. The four best categories are marked with asterisks.

**Fig 4:**
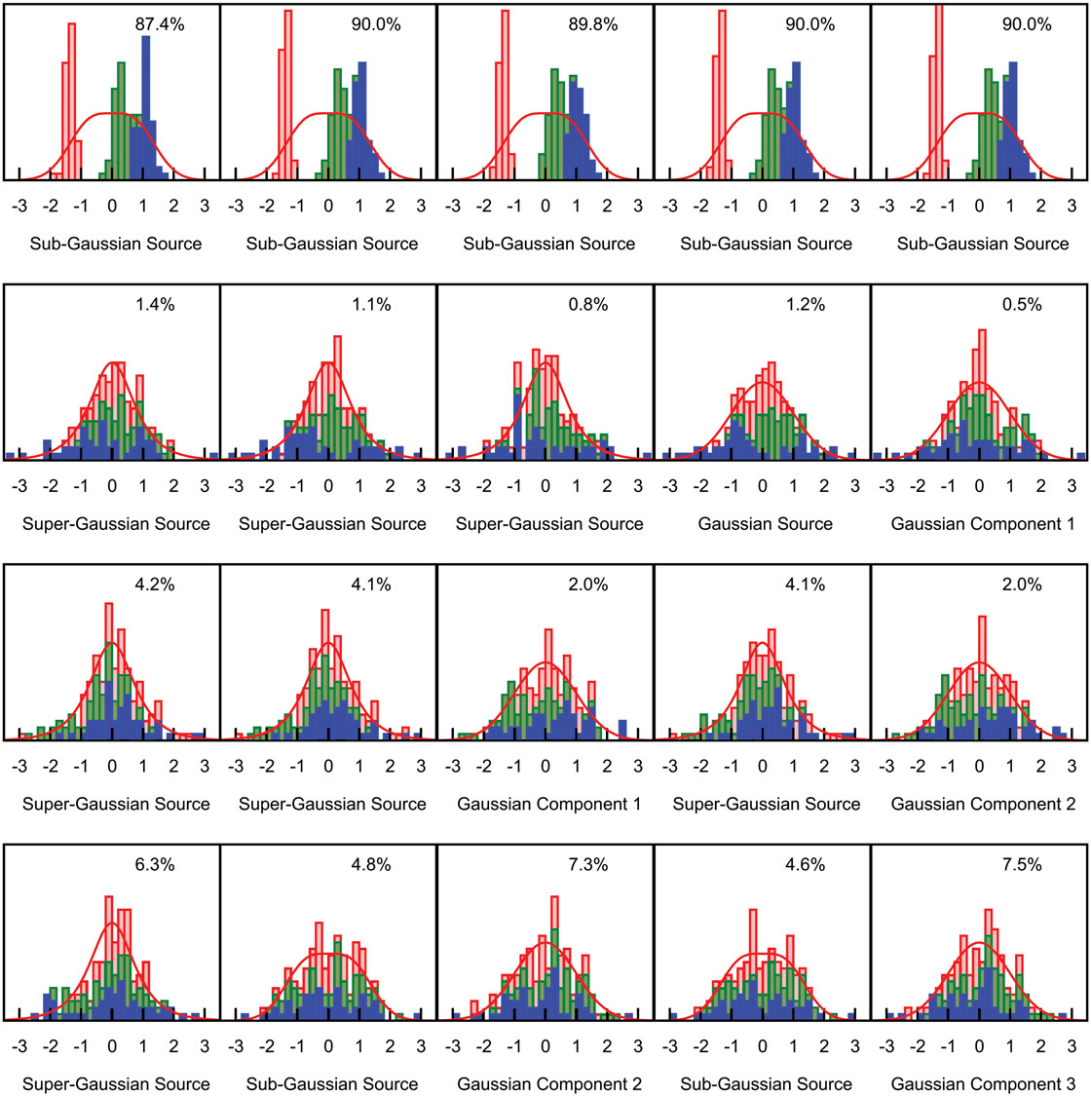
Distributional results for the five best model categories for the iris dataset. Histograms of sources for the best fitting model in each of the five best model categories are shown in columns in order of $\frac{n}{n-1}{\hat{\text{T}}}_{n-1}$ with the corresponding model source distribution superimposed. Each species is shown in a different color, *I setosa* in red, *I versicolor* in green and *I virginica* in blue. Histograms have been displaced vertically when necessary to prevent members of one species from obscuring those of another species. The non-Gaussian sources from each model have been sorted such that the most strongly correlated sources from the different models appear in the same row. The percentage values reflect the contribution of each source to the total variance.

Fig. 3 and Fig. 4 show mixed ICA/PCA results obtained from iris data originally analyzed by R.A. Fisher [21]. Noting that the iris species *I*. *setosa* is diploid, that the species *I*. *virginica* is tetraploid and that the species *I*. *versicolor* is hexaploid, Fisher was interested to test the hypothesis that *I*. *versicolor* might be a hybrid of the other two species, effectively two parts *I*. *virginica* and one part *I*. *setosa*. Using a linear discriminant function derived from sepal and petal widths and lengths derived from *I*. *setosa* and *I*. *versicolor* samples, he demonstrated that *I*. *versicolor* specimens had mean linear discriminant function scores that were indeed intermediate between those of the other two species. While species membership of each sample was required for derivation of Fisher’s linear discriminant function, here species membership was ignored when applying ICA/PCA, thus for this dataset, *m* = 4 and *n* = 150 (50 samples from each of three species). Fig. 3 illustrates the estimated values ${\hat{\text{T}}}_{n}=\frac{n}{n-1}{\hat{\text{T}}}_{n-1}$ based on cross-validation and the associated estimated bias of $\log\left[g\left(\underline{y}\right)|\hat{\underline{\theta }}\left(\underline{y}\right)\right]$ as an estimator of T_*n*_ for the fifteen possible categories of models for mixtures of four Gaussian, sub-Gaussian or super-Gaussian sources. Each model category was initialized 5000 times by applying random orthonormal rotation matrices to the data (the primary strategy of exchanging the columns of the identity matrix used to initialize **W** was also applied to each of the 5000 initializations). Sub-optimal local optima substantially worse that the best local optimum for the category were identified for as many as 80% of the initializations for some model categories, while other model categories invariably converged to the global optimum. Only results for the best initialization for each category are shown. The standard AIC estimate of the bias is also shown for each model. The model with the best support had one sub-Gaussian and three super-Gaussian sources, but four other model categories had values of ${\hat{\text{T}}}_{n}$ that were within 1.0 unit of this best model. All five of these model categories would therefore be classified as having similarly strong empiric support. Common to all five model categories was the inclusion of at least one sub-Gaussian source, and all model categories without at least one sub-Gaussian source had values of ${\hat{\text{T}}}_{n}$ substantially more than 5.0 units lower than the best model, indicating very poor empiric support. Thus the presence of a sub-Gaussian source is strongly supported. Four of the five best model categories also included at least one super-Gaussian source; the fifth had no super-Gaussian source and had a three dimensional Gaussian subspace. Fig. 4 shows the source distributions for each of the five model categories. The models are arranged in columns, and the sources for each model have been placed in rows such that the separable sources in each row are maximally correlated across categories. For categories having a Gaussian subspace with more than one dimension, PCA criteria were used to generate components, which were ordered by the amount of total variance explained. Although the ICA/PCA algorithm was blinded to species identities, these are illustrated in Fig. 4.

Fig. 4 shows that for all five of the best-fitting model categories, a sub-Gaussian source is generated (first row). The scores for this sub-Gaussian source were strongly correlated across all five categories, with all pairwise Pearson and Spearman correlations coefficients greater than 0.99. Thus support for a genuine, separable underlying source is very strong, as confirmed by the fact that the source scores allow almost complete separation of the three species.

The super-Gaussian sources generated by three models in the second row of Fig. 4 had Pearson correlation coefficients ranging from 0.85 to 0.93 and Spearman correlation coefficients ranging from 0.81 to 0.92. A Gaussian source in that row (separable as a source rather than a component since the corresponding model had a one dimensional Gaussian subspace) was also correlated with the three super-Gaussian sources; Pearson correlation coefficients with this Gaussian source ranged from 0.81 to 0.99, and Spearman correlation coefficients ranged from 0.76 to 0.99. Although the failure to identify this as a separable source in one of the five model categories (specifically, the model in the fifth column) raises some concern, and although the source is somewhat variable across the other four models, the data suggest that it would be worthwhile to try to understand the biological basis underlying this source even though it accounts for only a small part of the total variance (less that 1.5% across all five models).

The third row of Fig. 4 includes three more super-Gaussian sources, all with pairwise Pearson and Spearman correlation coefficients greater that 0.99. Thus despite concern as to whether they are truly separable due to lack of separation in two of the five models, the source represented in row three of Fig. 4 is highly consistent across those model categories that do attempt to separate them, indicating robustness to model misspecification.

The lowest reproducibility across models was seen for the sources in the fourth row of Fig. 4. Both Pearson and Spearman correlation coefficients ranged from. 79 to. 99 across the three models generating non-Gaussian sources. The poorest correlations were between the model that included a super-Gaussian source in this row and the two models that included a sub-Gaussian source. Given that this source was also not separated in two of the five models, it is the most suspect of the sources generated by ICA.

For the sub-Gaussian source on the first row of Fig. 4, additive genetic variance, as originally proposed by Fisher is a reasonable explanation since the hexaploid hybrid (mean score 0.31, standard deviation 0.22) has scores that are intermediate between the diploid (mean score -1.38, standard deviation 0.12) and tetraploid (mean 1.06, standard deviation 0.22) species but closer to those of the tetraploid. The super-Gaussian source on the second row of Fig. 4 appears to be driven by a bimodal distribution within the *I*. *virginica* specimens. The super-Gaussian source on the third row of Fig. 4 reflects differences between species where the hexaploid *I*. *versicolor* (mean -0.54, standard deviation 0.88) is not intermediate between *I*. *setosa* (mean 0.24, standard deviation 0.96) and *I*. *virginica* (mean 0.30, standard deviation 0.89). The hybridization event that created *I*. *versicolor* is now thought to have involved an *I*. *setosa* specimen more closely related to the variety indigenous to the Alaskan interior than to the Canadian variety used for Fisher’s analysis [22], so genetic factors within the data set beyond simple hybridization are plausible. Environmental factors may also contribute to either or both of the super-Gaussian sources since Fisher noted that the *I*. *setosa* and *I*. *versicolor* specimens were found growing together in the same colony while the *I*. *virginica* specimens were acquired from a different location. Species membership does not give strong support to any of the alternative classifications of the sources or components illustrated in the fourth row of Fig. 4, suggesting either a genuine source influencing all three species similarly or the absence of a fourth identifiable non-Gaussian source.

### Example 2: Human Craniometric Measures

**Fig 5:**
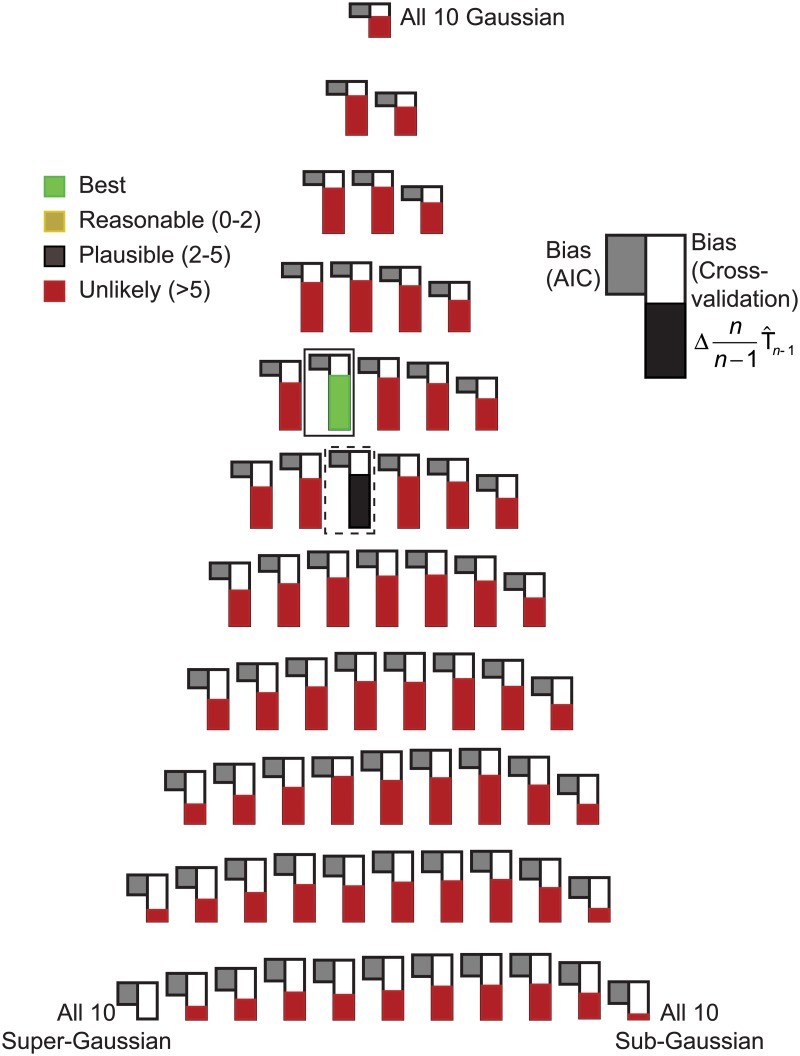
Cross-validation results for the craniometric dataset. For each of the 66 model categories, the best fitting model within the category is shown. The solid bar in color on the right represents the increase in $\frac{n}{n-1}{\hat{\text{T}}}_{n-1}$ relative to the poorest fitting category, and the solid white bar above it represents the estimated bias in the optimized log-likelihood of the model as an estimate of T_*n*_. The bar on the left shows the AIC bias estimate based on the number of freely adjustable parameters in the model. The best fitting model is enclosed by a solid box. The dashed box encloses the model category that included the second and third best fitting models.

**Fig 6:**
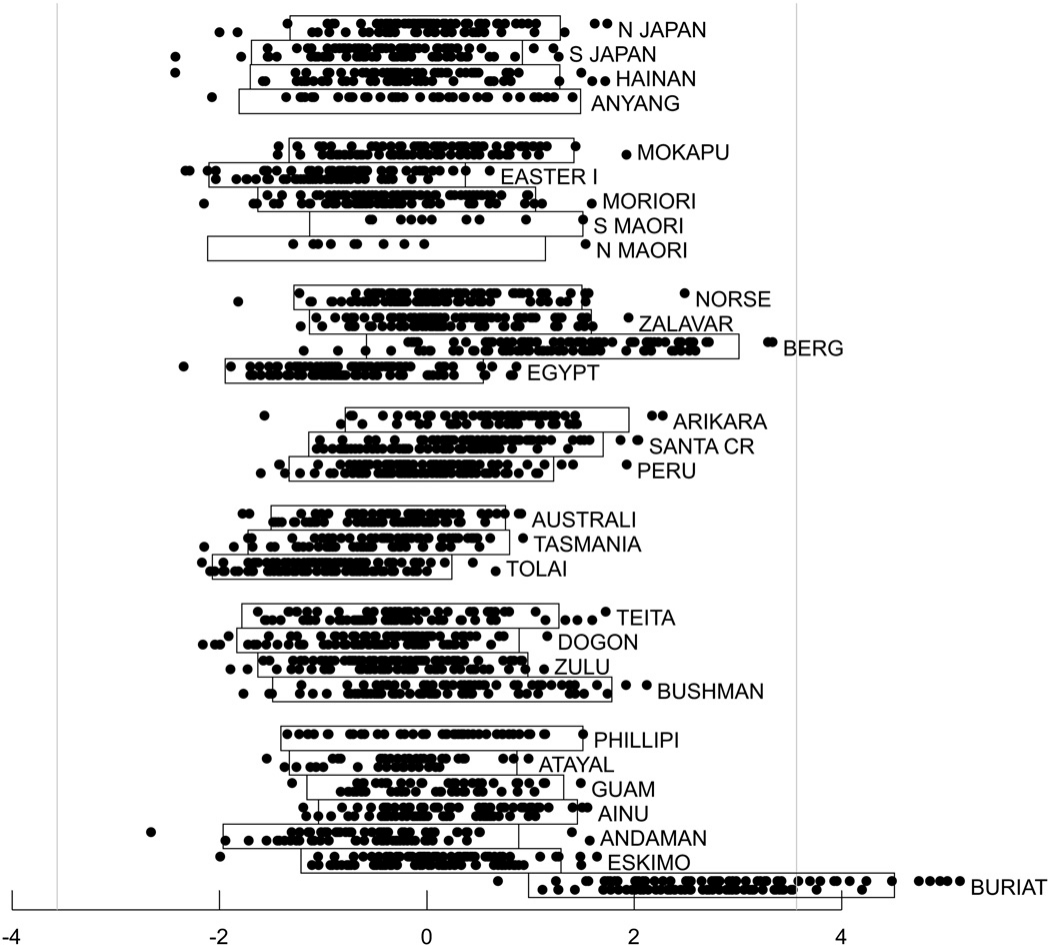
Super-Gaussian source separating the Buriat population from other populations. Source scores are derived from the best fitting model. This source accounts for 7.4% of the variance of the original data set. All members of each of the thirty populations are plotted on the same subpanel with males at the top of the subpanel and females at the bottom. The rectangular boxes enclose individuals with source scores within two standard deviations of the population mean. The vertical light gray lines are based on the observed mean and standard deviation for the entire population of 2524 subjects and positioned such that just one subject would be expected to fall outside those lines if the distribution were Gaussian. Populations are grouped according to Howells’ six main groups (Far Eastern, Polynesian, European, American, Austro-Melanesian and African), with Howells’ seven ungrouped populations together at the bottom.

**Fig 7:**
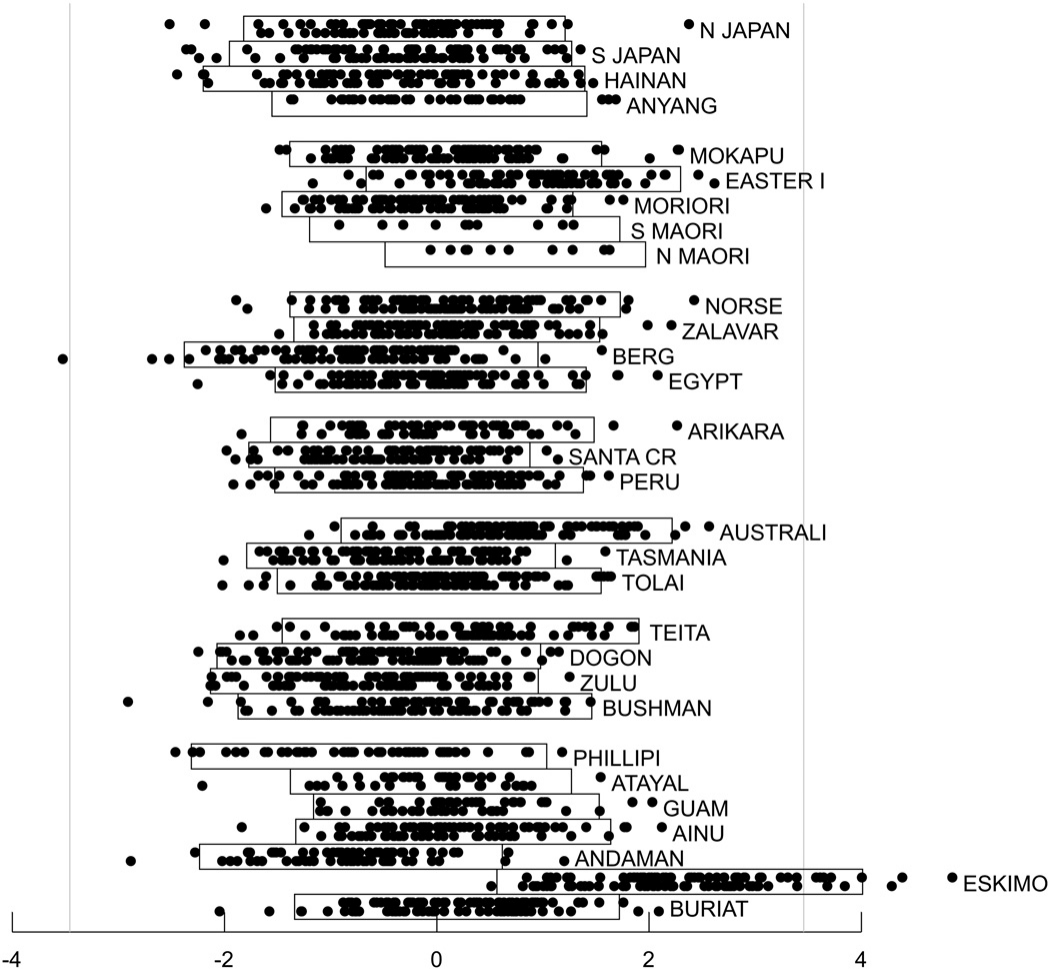
Super-Gaussian source separating the Eskimo population from other populations. Source scores are derived from the best fitting model. This source accounts for 6.4% of the variance of the original data set. See Fig. 6 legend for additional details.

**Fig 8:**
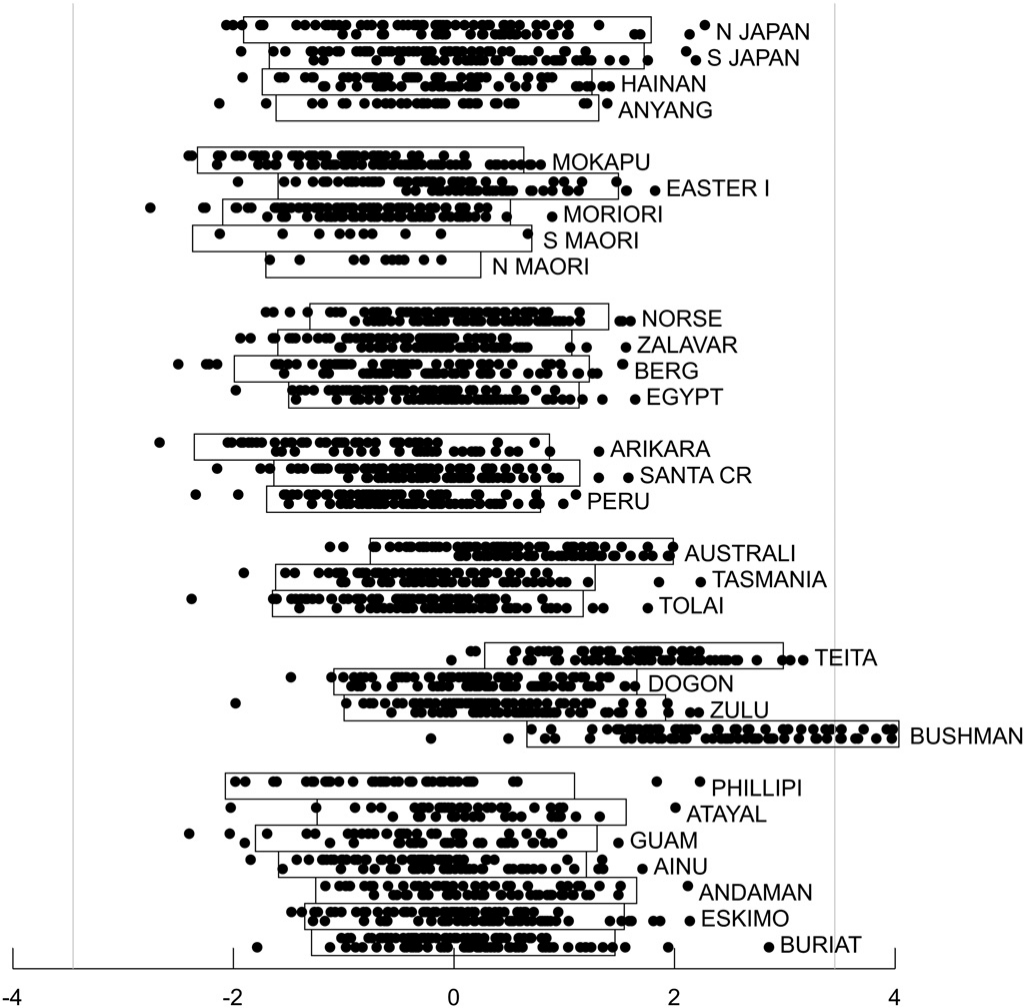
Super-Gaussian source separating the Bushman and Teita populations from other populations. Source scores are derived from the best fitting model. This source accounts for 18.0% of the variance of the original data set. See Fig. 6 legend for additional details.

**Fig 9:**
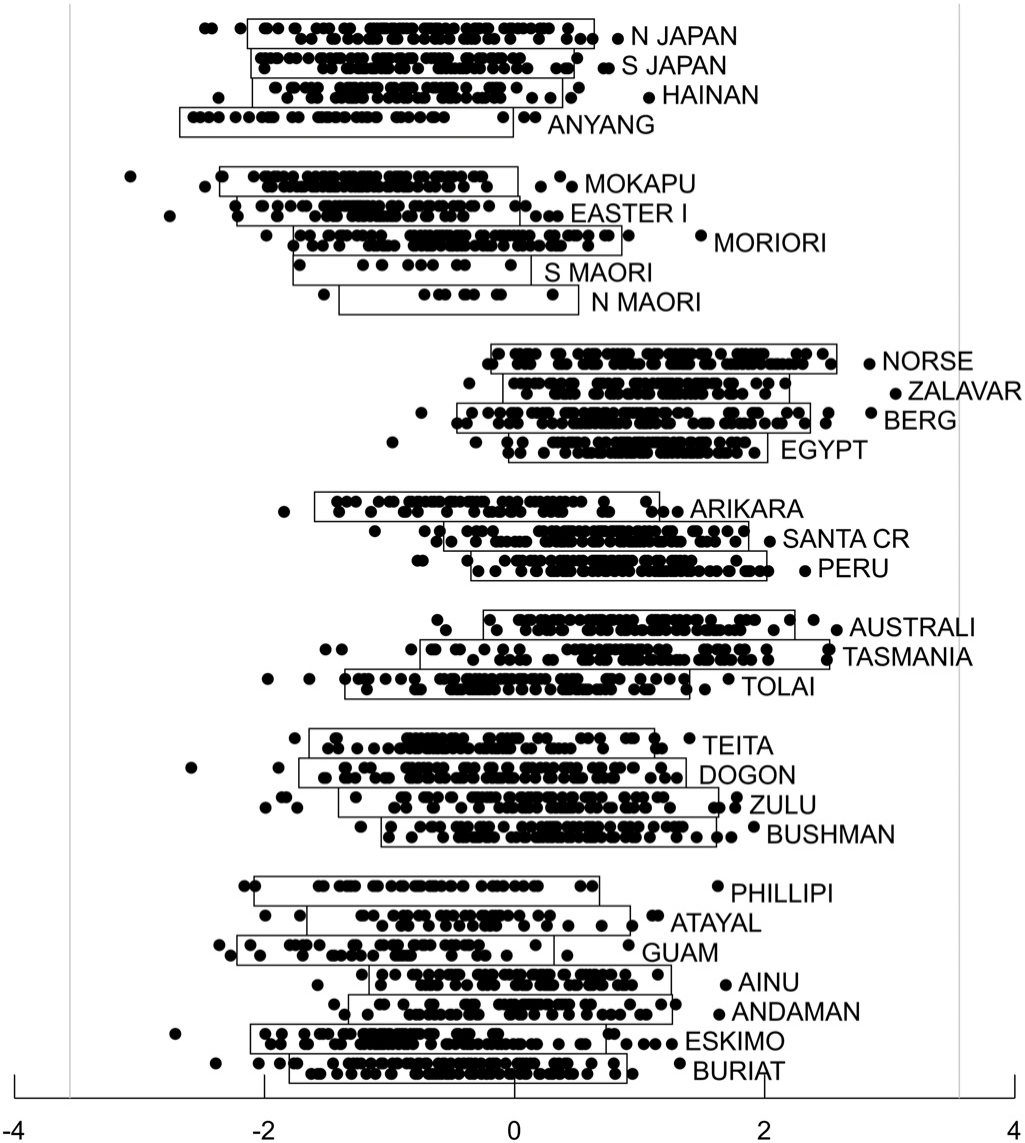
Sub-Gaussian source separating the Far Eastern group and the European group from one another. Source scores are derived from the best fitting model. This source accounts for 10.0% of the variance of the original data set. See Fig. 6 legend for additional details.

**Fig 10:**
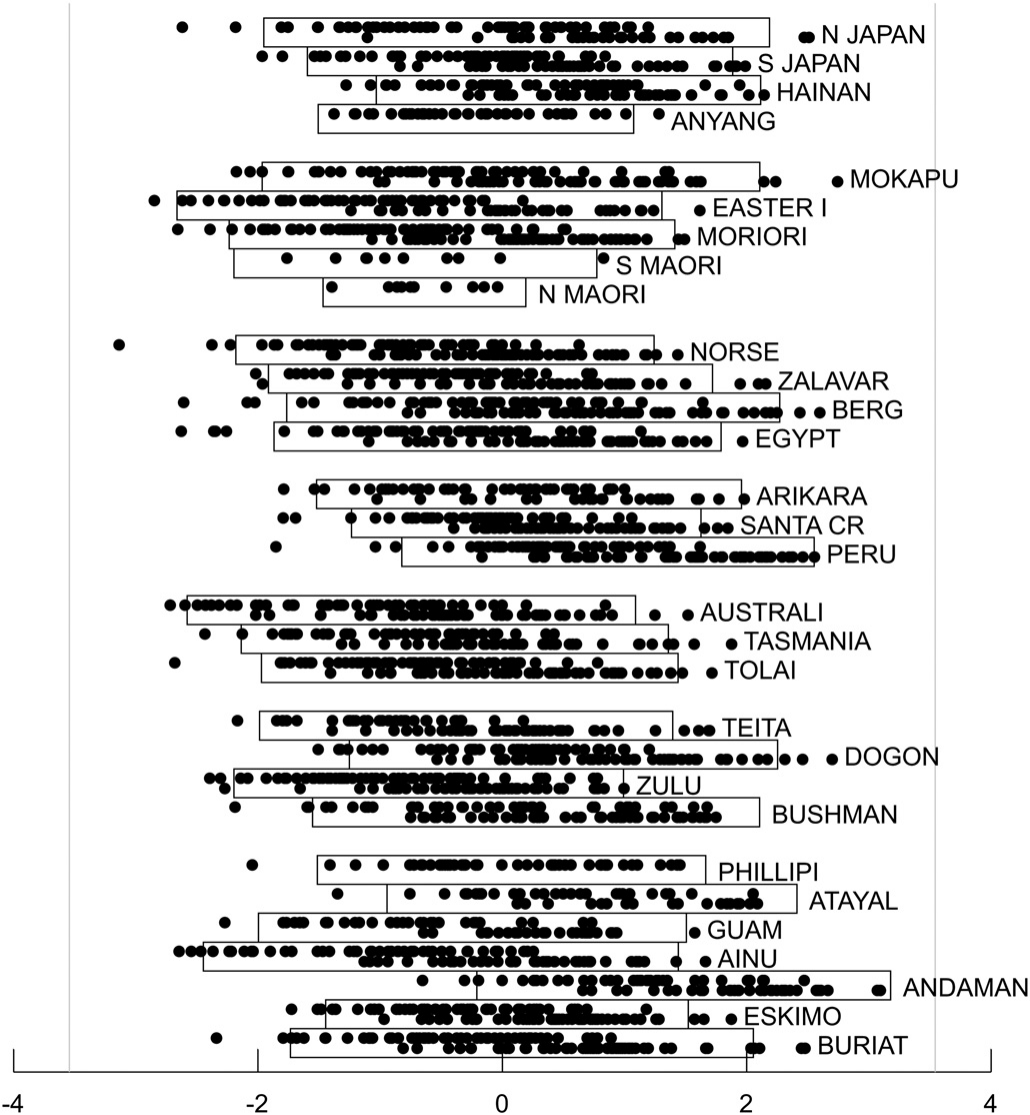
First principal component of the craniometric data six dimensional Gaussian subspace. Component scores are derived from the best fitting model. This component accounts for 20.0% of the variance of the original data set. See Fig. 6 legend for additional details.

Figs. 5 to 10 show craniometric data derived from 2524 human crania from thirty different human populations collected by Howells [23], [24], [25], [26]. Howells grouped twenty-three of the populations into six major categories: Far Eastern (North Japan, South Japan, Hainan and Anyang); Polynesian (Mokapu, Easter Island, Moriori, Southern Maori and Northern Maori); European (Norse, Zalavar, Berg and Egypt); American (Arikara, Santa Cruz and Peru); Australo-Melanesian (Australia, Tasmania and Tolai) and African (Teita, Dogon, Zulu and Bushman). These assignments are largely in accord with more recent groupings based on DNA analyses [27], [28], [29] except that Polynesian populations are now thought to primarily derive from aboriginal Taiwanese who likely originated in Southern China, which would make them a subset of the Far Eastern group, but also have varying degrees of Australo-Melanesian admixture [30], [31]. Howells did not classify seven additional populations (Philippine, Atayal, Guam, Ainu, Andaman Islands, Eskimo, and Buriat) as belonging to a particular group. The data were originally made publicly available in electronic format by Howells in 1996 [26] and were accessed for the work described here at http://web.utk.edu/~auerbach/HOWL.htm.

The original data set included 82 different cranial measurements. Only seventy-one of these measurements were available for all 2524 specimens; the other eleven measurements were therefore discarded for the current analysis. Two separate sets of analyses were performed. For the first set of analyses, PCA was used to reduce the original 71 by 2524 centered observation matrix $\tilde{X}$ to a smaller 10 by 2524 matrix (*m* = 10, *n* = 2524), which was then subjected to the complete mixed ICA/PCA procedure described above. The 10 retained components accounted for 83.6% of the variance in the full data set. The choice to retain ten dimensions was based on a desire to keep computation times manageable for illustrating use of the mixed ICA/PCA algorithm and was not a data driven decision. Since the raw measurements included a mixture of both distances and angles, which are therefore represented in different units, reducing the number of dimensions based on raw measurement variances would not normally be the recommended approach (for example, one might instead prefer to rescale the angular measurements such that their average variance matches that of the distance measures), but is sufficient for purposes of illustration. In the second set of analyses, all seventy-one measurements were used without any dimensionality reduction, and various strategies described above for high dimensional data were used to identify a best known model, with the caveat that substantially better models may exist but remain unknown. In all instances, the ICA/PCA algorithm was blinded to the population from which specimens were derived and to the sex of the specimens. To the extent that operator dependent decisions factored into the analysis of the non-reduced data, the operator made these decisions without knowledge of subject population or sex.

For the ten sources in the PCA reduced dataset, each of which can be sub-Gaussian, Gaussian or super-Gaussian, a total of 66 model categories involving differing numbers of each type of source can be considered. To evaluate the effectiveness of the primary strategy for avoiding local minima by permuting the initial assignment of each type of source to each of the ten rows of the centered and PCA reduced observation matrix, all 66 model categories were rerun twenty additional times, each with a different and unique random orthonormal rotation matrix **O**_*R*_ applied as a secondary strategy for avoiding local minima as described above. In several instances where eight, nine or all ten of the sources were modeled as being the same type, some disagreement was observed among the twenty-one different initializations, but with one exception, disagreement was not observed when no more than seven of the sources were of the same type. It should be noted that the primary strategy for avoiding local minima provides far fewer initializations when most or all sources are of the same type (e.g., only one initialization when all are identical) than when types are mixed. With the one noted exception, disagreements among initializations only occurred when the primary strategy employed 45 or fewer initializations. Mixed ICA/PCA models that had nine or more sources of the same type were therefore all run 100 additional times with new unique random orthonormal rotation matrices; in all instances, the best optimum identified from the initial twenty-one initializations remained the best optimum over the full complement of 121 optimizations. The one exception mentioned above was for a model category with three sub-Gaussian, five super-Gaussian and two Gaussian sources; in this instance 19 of the 21 initializations produced final bias corrected loglikelihoods that were 1.33 to 7.49 units smaller than the best initialization.

For each of the 66 model categories, Fig. 5 shows the cross-validation estimate of the bias when the optimized log likelihood is used as an estimate of T_*n*_. Model categories shown on the same row have the same number of Gaussian sources and therefore share the same AIC bias estimate, which is also shown in the figure. As anticipated from the simulation studies, the cross validation estimate of the bias is considerably larger than the AIC estimate when non-Gaussian sources are modeled. When no Gaussian sources were included (corresponding to traditional ICA), the bias varied by more than 30 units depending on the specific numbers of sub-Gaussian and super-Gaussian sources even though the number of free parameters in these non-Gaussian models were all identical.

After using cross-validation to correct the bias of the optimized log likelihood, the best fitting model included one sub-Gaussian source, three super-Gaussian sources and a six-dimensional Gaussian subspace (solid box in Fig. 5). To arrive at this particular model, cross validation was used to estimate and correct for bias of over 200 distinct local optima across the 21 random initializations for this category. The magnitude of bias varied substantially (more than 120 units) across different optima for this single model category. The four non-Gaussian sources were nearly, but not precisely orthogonal to one another, deviating from orthogonality by 0.2 to 0.9 degrees; the Gaussian components were precisely orthogonal to each other and to the non-Gaussian sources. The second best fitting model included two sub-Gaussian sources, three super-Gaussian sources and a five dimensional Gaussian subspace (dashed box in Fig. 5) and differed from the best fitting model by 4.86 units indicating very weak empirical support for preferring this model over the best fitting one. The third best fitting model also had two sub-Gaussian sources, three super-Gaussian sources and a five dimensional Gaussian subspace and is not shown in Fig. 5 because it was a distinct local minimum rather than the global minimum for this source combination category. It differed from the best fitting model by 10.84 units; the reason for mentioning this unlikely model will be clarified below. All other models differed from the best fitting model by more than 13 units, indicating, in the absence of any prior knowledge, virtually no empirical support for preferring these models over any of the best three.

Fig. 6 and Fig. 7 show the source scores for two of the super-Gaussian components from the best fitting model for all 2524 individuals, grouped by population and sub-grouped by sex. Both of these sources were driven outlier populations, in one instance (Fig. 6) the Siberian Buriats (and to a lesser extent the Austrian Berg) and in the other case (Fig. 7), the Greenland Eskimos. These two sources were particularly robust and stable across the 66 different source categories considered. For every category that included at least one super-Gaussian source, a super-Gaussian source highly correlated with the Buriat source shown in Fig. 6 was identified in the best fitting model for that category (Pearson and Spearman correlations greater than 0.95). Similarly, for every category that included at least two super-Gaussian sources, a super-Gaussian source highly correlated with the Eskimo source shown in Fig. 7 was found (Pearson and Spearman correlations greater than 0.96). Thus these two sources are both very robust to model misspecification. Models with less than two super-Gaussian sources invariably fit poorly as compared to other models, so the empirical evidence in support of these two sources is very strong.

Also from the best fitting model, Fig. 8 shows an additional super-Gaussian source that is driven by African populations, particularly Bushman and Teita as outliers. Fig. 9 shows a sub-Gaussian source that almost completely separates the European group from the Far Eastern group. The Gaussian component accounting for the most variance in the six dimensional Gaussian subspace is shown in Fig. 10. The second and third best fitting models both included all four of the non-Gaussian sources found in the best fitting model (Pearson and Spearman correlation coefficients 0.98 or greater). The additional sub-Gaussian source added by the second best fitting model incompletely separated the European and Australo-Melanesian groups, with other populations lying intermediate (not shown). While plausible, this source will not be discussed further given the very weak empirical support for this model. The additional sub-Gaussian source added by the third best fitting model was clearly driven by differences between males and females (not shown). Sex did not emerge as a non-Gaussian source in either of the two best fitting models. However, as seen in Fig. 10 for the best fitting model, sexual dimorphism was clearly evident in the first principal component of the Gaussian sub-space. Pearson and Spearman correlation coefficients between the sex-associated non-Gaussian source from the third best fitting model and the sex-associated Gaussian component from the best fitting model were 0.82 and 0.81 respectively.

For the full craniometric data set analyzed without dimension reduction, the best identified model included one sub-Gaussian source (shown in S1 Fig), forty super-Gaussian sources (sorted by descending degrees of associated variance in S2–S41 Figs) and a thirty dimensional Gaussian subspace (components, sorted by descending degrees of variance in S42–S71 Figs). No other models had log likelihoods sufficiently close to this model to be considered plausible, but it is possible that other plausible or even better models exist but were not identified. In addition to the Buriat (S4 Fig) and Eskimo (S9 Fig) driven sources already known from the reduced dimension modeling, several other super-Gaussian sources were clearly driven by single populations including the Moriori (perhaps in combination with the N and S Maori) (S8 Fig), the Easter Islanders (S11 Fig), the Santa Cruz (S12 Fig), the Andaman Islanders (S14 Fig), the Australi (S16 Fig), the Ainu (S20 Fig), the Bushman (S22 Fig), the Tasmanians (S24 Fig), and the Dogon (with some outliers in other populations) (S29 Fig). Groups of populations or opposing pairs or groups of populations clearly drive several other sources. Many of the super-Gaussian sources were driven by outliers from several different populations, including sources that accounted for some of the largest amounts of variance. The combined Bushman and Teita source from the data reduced to ten dimensions shown in Fig. 8 did not have a strict counterpart in the full data analysis, instead splitting into the Bushman source shown in S22 Fig and the source shown in S2 Fig, which is driven in part by the Teita in one direction and by various European outliers in the opposite direction. The sub-Gaussian source identified in the reduced data set was distinct from the sub-Gaussian source from the full data set and correlated best (but not strongly) with the super-Gaussian sources shown in S5 Fig and S6 Fig. The sub-Gaussian source from the full data set instead showed strong sexual dimorphism (S1 Fig). While correlated with overall size differences, sexual dimorphism is well documented in the literature [32].

**Fig 11:**
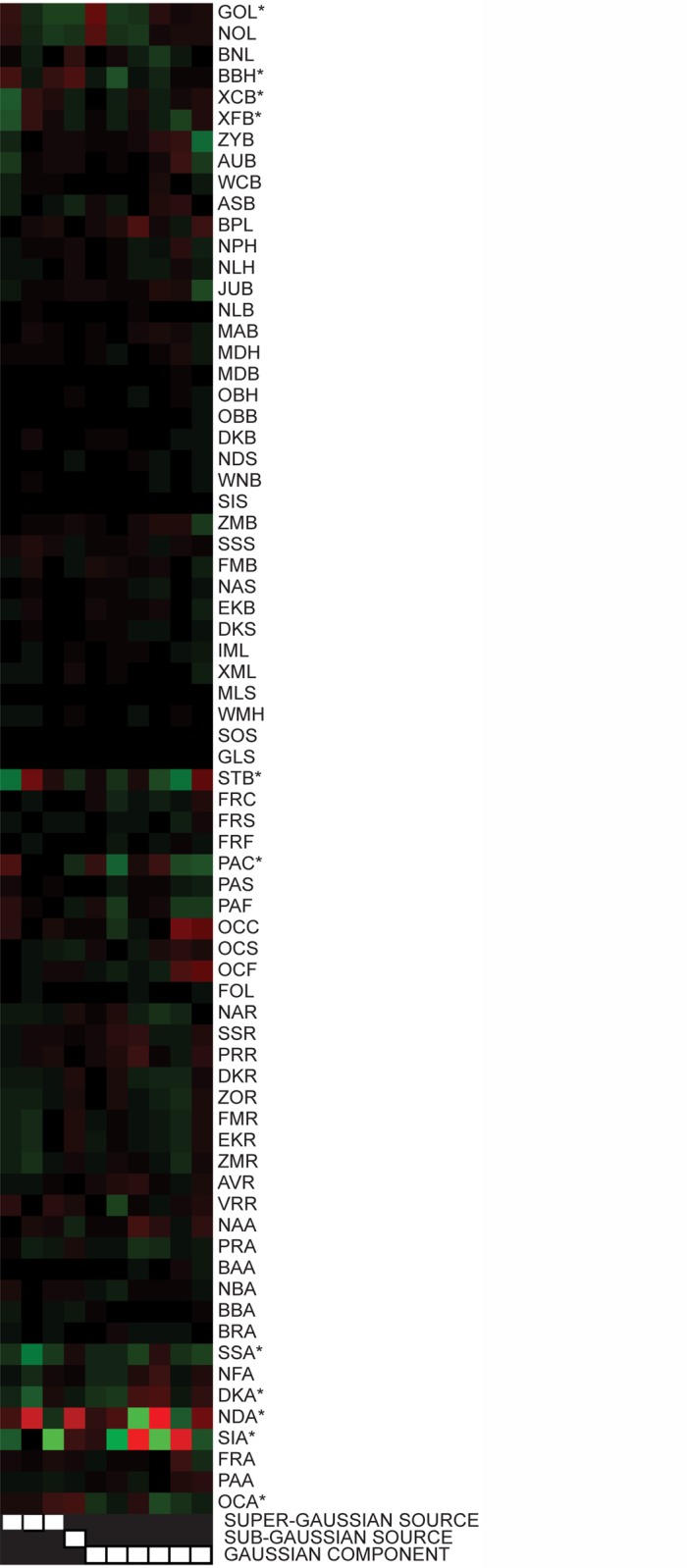
Heat map relating z-scores to sources and components for the craniometric data. Heat map intensities are derived from the best fitting model. Bright green values indicate that a higher (more positive) z-score contributes to a positive source or component score. Bright red values indicate that a higher (more positive) z-score contributes to a negative source or component score. The first four columns correspond sequentially to the sources shown in Figs. 6–9. The fifth column corresponds to the first principal component shown in Fig. 10. The last four columns correspond to components accounting for increasingly smaller amounts of total variance (not shown). Raw data measures marked with asterisks are prominent contributors to the identified non-Gaussian sources and are discussed in the text.

Fig. 11, derived from the data set reduced to ten dimensions, illustrates how the matrix **W** can be used to characterize the raw data measures most prominent in defining individual sources. Right multiplying **W** by the transpose of the left singular vectors obtained from the initial singular value decomposition used to reduce the rows of the observation matrix from 71 to 10 produces a new 10 by 71 unmixing matrix that can be applied directly to the 71 by 2524 matrix $\tilde{X}$ to generate the sources. If the rows of $\tilde{X}$ are then individually rescaled by their standard deviations to create a matrix of *z*-scores, the corresponding columns of this new unmixing matrix can be adjusted by the same factors to preserve the multiplicative mathematical relationship to the sources. For the best-fitting model, Fig. 11 shows a heat map reflecting how the z-scores for each of the original 71 measures are weighted in generating each of the four ICA sources and six PCA components. A comparable heat map for the full data set without dimension reduction is shown in S72 Fig.

From Fig. 11, it is evident that several different measures contribute prominently to the super-Gaussian source separating the Buriat population (Fig. 6) from others. The naso-dacryal angle (NDA), roughly the angle formed by the sides of the nose at the upper margin of the orbits, is narrow, while the simotic angle (SIA), a similar measure made further down on the nose, is large in the Buriates. Both the height of the skull measured from bregma to basion (BBH) and the midline length of the parietal bone (PAC) are comparatively smaller, while the frontal breath of the skull (STB, XCB, and XFB) is comparatively larger. Roseman [33] has suggested that this brachycephalization observed in the Buriat sample is due to the effect of natural selection operating in the cold Siberian environment, while acknowledging that it is not observed in the Eskimo sample. Given that an increase in brachycephalization has been reported as a result of recommendations that infants sleep on their backs to reduce the risk of sudden infant death syndrome [34], an environmental contribution related to childrearing practices during infancy might also be considered.

For the super-Gaussian source separating the Eskimo from other populations (Fig. 7), a narrow naso-dacryal angle is a particularly strong contributor. Frontal breath as quantified by bistephanic breadth (STB) is comparatively smaller in the Eskimo. A large zygomaxillary angle (SSA) and dacryal angle (DKA), both measures of flatness of the face also contribute to this source.

The super-Gaussian source with the Bushman and Teita populations as outliers (Fig. 8) is very heavily influenced by a large simotic angle (SIA) in these populations, with the Bushman population having the largest simotic angles among all populations sampled. A comparatively larger glabello-occipital length (GOL), reflecting a larger length of the skull and smaller occipital angle (OCA), reflecting a flatter occipital region, are more minor contributors favoring membership in the Bushman or Teita populations.

For the sub-Gaussian source that separates the Far Eastern group from Europeans (Fig. 9), a larger naso-dacryal angle (NDA) and simotic angle (SIA) across the nose, a larger bregma to basion height (BBH), and a larger occipital angle (OCA) are characteristic of the Far Eastern group, while those in the European group have a larger glabello-occipital length (GOL). Thus the nose is sharper and the skull shorter and more elongated in the European group relative to the Far Eastern group.

Comparison of Fig. 11 to the corresponding columns in S72 Fig indicates that many other measures contribute to the separation of the Buriat and Eskimo populations than were evident from the reduced data set, and S72 Fig also provides insights into defining characteristics of the other populations that can be separated in the full data set. Of particular note are contributors to the two super-Gaussian souces (S40 and S41) explaining the smallest amounts of variance. Each of these is driven by a different set of three measures. In each instance, the three measures were the angles of a triangle defined by three landmarks, and since the angles of a triangle always sum to 180 degrees, the measures are theoretically not linearly independent. Inspection of the original data confirmed that due to round-off errors to the closest whole degree, the sum of the three angles values was always either 179 degrees, 180 degrees or 181 degrees, with the rounding process being the underlying “source” in both cases. Future analyses of the craniometric data set should obviously omit the third angle in each case.

A complete listing of the coefficients required to compute the four non-Gaussian source scores and the six Gaussian component scores from the 71 raw measures is included in S1 Table, along with the standard deviations of the raw measures needed to convert the coefficients into the heat map shown in Fig. 11. S2 Table contains analogous data for the forty-one non-Gaussian source scores and thirty Gaussian component scores for the analysis without dimension reduction.

## Discussion

The mixed ICA/PCA model described here can be viewed as a special instance of a class of ICA techniques that explicitly model more than one source distribution as part of the ICA optimization [10], [11], [35], [36]. Although multiple Gaussian sources are not separable from one another using ICA, this does not preclude their inclusion in the ICA model. Indeed, in previous work, Attias [35] included an isolated Gaussian source in addition to Gaussian mixtures in an ICA model. However, the incorporation of Gaussian sources into the model introduces a model selection problem not considered in conventional ICA, which is related to the reduced number of parameters needed to characterize Gaussian components, an issue not addressed by Attias’ Gaussian mixture formulation. Unfortunately, assumptions underlying the Akaike Information Criterion for model selection are not valid when inseparable Gaussian components are modeled as non-Gaussian sources, rendering this well-known approach to model selection in the setting of differing model complexity unreliable. As a result, the computationally expensive cross-validation approach of Stone [19] has been adopted here to correct for biases in the estimation of K-L divergence from truth. From a practical standpoint, the fact that each Gaussian source must be orthogonal to all other sources when the likelihood is maximized simplifies the ICA optimization problem that must be solved.

PCA is often used as a preprocessing step in traditional ICA analysis, making it relevant to distinguish such preprocessing from the approach described here. When used for preprocessing, the data are centered, PCA is applied, a certain number of components explaining the smallest amounts of variance are discarded and ICA is then applied to the remaining components. The discarded components are therefore Gaussian and orthogonal to one another and to all ICA-derived sources, just like Gaussian components derived by mixed ICA/PCA. However, this preprocessing approach ignores the possibility that the discarded low-variance components may nonetheless contain separable non-Gaussian signals. The mixed ICA/PCA algorithm offers a more general approach; the possibility that these low-variance components might not contain any non-Gaussian signals is included in the mixed ICA/PCA modeling, but only as one of a large number of alternative solutions. The K-L divergence from truth of a mixed ICA/PCA derived model without preprocessing should always be less than or equal to the K-L divergence of a model that uses PCA preprocessing since the number of adjustable parameters for a given number of Gaussian components is the same and the preprocessing model is a nested model encompassed by the more general mixed ICA/PCA model. As illustrated by the craniometric data set, some degree of PCA preprocessing to reduce data dimensionality can substantially decrease computation time, potentially making it a pragmatic complement to subsequent mixed ICA/PCA analysis when the number of potential sources is large. When PCA preprocessing is used in this way, it is advantageous to retain as many dimensions as is practical for analysis using mixed ICA/PCA so as to minimize the risk of discarding non-Gaussian signals.

Aside from choosing between super-Gaussian and sub-Gaussian sources, the issue of model selection in ICA has received little attention. The practical impact of the number of components discarded through PCA preprocessing in traditional ICA has been recognized [37], [38], [39] and the use of AIC, BIC and other information criteria has been described in this setting [40], [41], but this does not address the issue of a potentially inseparable Gaussian sub-space in the retained data dimensions. Model selection has also been considered in the context of blind-source separation of time series where columns in **X** correspond to measurements made at different time points [42], [43], [44], but this is a distinctive problem domain where separation of mixtures of Gaussian sources is sometimes possible [3], [40], [45], making the model selection issues described here less critical. It should be noted that the requirement that the *n* multivariate observations be independent would be violated if the methods described here were used for temporal or spatial correlations among the *n* observations, resulting in an under-correction of bias in the cross validation step. If one nonetheless wanted to use the framework described here for temporally or spatially dependent data, this could be approached by subsampling the data based on the autocorrelation length to produce samples that are effectively independent, an approach that has been previously described in the context of PCA preprocessing for traditional ICA [41].

Although not formally formulated as a model selection technique, recent work by Yang et al, [46] includes features that may empirically flag ICA sources that would be more appropriately considered as part of a Gaussian sub-space. Using the full data set, they randomly initialize the ICA algorithm multiple times and then rank the derived sources based on their reproducibility across initializations. When a Gaussian sub-space with two or more dimensions is present, the ICA “sources” spanning this subspace should become oriented randomly within it as sample size increases to infinity, leading to particularly poor reproducibility across initializations. However, as illustrated by both the iris data and the craniometric data, the best set of parameters for a given model does not necessarily correspond to the most frequently identified optimum across a set of random initializations, indicating the potential for inappropriately rejecting the best parameters simply because the global minimum cannot be reached by gradient descent from most random locations in parameter space. Conversely, for finite sample sizes, it is possible that a single global minimum might be reachable from any initialization of parameters but that the location of the minimum might be determined by noise that would not be reproduced by a new independent set of observations. A systematic evaluation of the specificity and sensitivity of the approach described by Yang et al. relative to the methodology detailed here would be of pragmatic interest.

Local maxima in the computed log-likelihoods are a significant issue in ICA, even when all sources are modeled as being from the same distribution using traditional ICA, and the problem is compounded when multiple types of distributions are modeled, breaking symmetries that would otherwise make certain initializations redundant. The primary strategy of systematically permuting the relationship between the rows of $\tilde{X}$ and the columns of the initial identity matrix **W** was not universally sufficient to identify global maxima that were identifiable by the secondary strategy of left multiplication of $\tilde{X}$ (mathematically equivalent to right multiplication of the initial identity matrix estimate of **W**) by random orthonormal rotation matrices. Consequently, confirmatory use of the secondary strategy of random rotations is recommended, particularly when the primary strategy does not lead to a large number of distinct initializations. It could be argued that even random orthonormal rotations risk missing a global maximum that might be identified by a completely random initialization of **W**, but unlike orthonormal matrices, random matrices or even unitary matrices do not form a compact group, so pragmatic limits would need to be placed on the range of determinants and the degree of anisotropy allowed for the initial value of **W** in any case, and the number of required initializations, already a major limitation, could easily become impractical. It is impossible to assure that the global maximum has been found, so the decision of how exhaustively to search is ultimately a pragmatic one.

It is important to emphasize that while mixed ICA/PCA may blindly identify group membership or salient explanatory variables that are already known or suspected in a multivariate data set thereby providing strong support for the role of these suspected factors, failure to confirm a suspected source of variance using this technique should not be construed as a “non-significant” test. Multivariate hypothesis testing and model selection techniques are available that are far more powerful, in part because they do not depend on non-Gaussian features and can therefore operate effectively within a non-separable Gaussian subspace.

The $\sqrt{n}$ term empirically observed in the simulation studies when mixtures of Gaussian sources are modeled as non-Gaussian raises the interesting question of whether this term persists as *n* goes to infinity. If so, this would imply that the problem is not an issue that might be addressed through derivation of some form of sample size correction for mixed ICA/PCA, but rather a more fundamental violation of the asymptotic assumptions used to formulate the AIC. If the $\sqrt{n}$ term does persist to infinity, it would also outpace the $K\frac{\log(n)}{2}$ correction associated with the BIC, so the issue of whether BIC would asymptotically afford consistent model selection is of concern as well. A key issue that may undermine the asymptotic validity of both AIC and BIC is the fact that when the underlying sources are mixtures of Gaussian distributions, the estimated ICA decomposition of those sources will be random and therefore will not converge to any fixed set of values as *n* increases to infinity. The possibility of asymptotically non-convergent model parameters is not a feature of the derivation of AIC or BIC, so both are suspect as bases for model selection in ICA/PCA.

The cross-validation work described here unexpectedly demonstrates that bias in the model selection problem is also relevant to conventional ICA if the end goal is considered to be minimizing K-L divergence from truth. Bias in the optimized log likelihood as a measure of T_*n*_ varies for differing numbers of sub-Gaussian and super-Gaussian sources even in the absence of Gaussian sources, and varies substantially for different local minima within a given model. The magnitude of this variation exceeds the differences in optimized log likelihood typically considered to strongly favor one model over another in other model selection contexts, suggesting that cross-validation would improve ICA interpretation even when no Gaussian sources are present.

The computational burden is also large as a result of the need to use cross-validation for model selection. Compared to simply computing the optimized likelihood of a particular model, an *n*-fold increase in computation is required. A computationally efficient alternative method for addressing the $\sqrt{n}$ bias term identified in the simulation studies when Gaussian components are modeled as non-Gaussian would be tremendously beneficial. However, it is clear that a simple adjustment to the optimized log-likelihood will not suffice since the decision of when to apply such an adjustment would require already knowing whether the sources were Gaussian or non-Gaussian. Recent developments in estimating bias in multivariate regression, which is equivalent to ICA/PCA with all sources being Gaussian, offer some hope that this problem might nonetheless be tractable. By adjusting the formula for the optimized log-likelihood itself, Yanagihara, Kamo and Tonda [47] were able to eliminate the bias term involving the multivariate kurtosis of the underlying distribution even though the underlying distribution is unknown. Unfortunately, the derivation of this adjusted formula involves equalities that do not generalize to the broader context of ICA, but future work might identify an alternative formula that does.

The specific sub-Gaussian and super-Gaussian distributions modeled by mixed ICA/PCA presumably may have an effect on whether sources are separable, a topic that warrants further investigation. For example, use of sources distributions that are extremely close to Gaussian would presumably require a larger value of *n* to demonstrate superiority to a Gaussian model. However, this might come at the advantage of being able to eventually (with sufficiently large *n*) identify very subtle non-Gaussian sources that might be not separable at any sample size when using non-Gaussian sources that are strongly non-Gaussian. Inclusion of parameterized non-Gaussian source distributions [11], [35] might be advantageous in allowing the modeled distribution to optimally adapt to the data, though potentially at the cost of increased difficulties with local optima. To the extent that specific distributions of potential non-Gaussian sources are known in advance, incorporating this information into the ICA/PCA model might help to optimize the ability to separate sources when sample sizes are small.

## Conclusions

In some sense, the model selection problem in ICA/PCA can be seen as analogous to the problem of deciding how many components to retain in PCA. Indeed, from a modeling perspective these problems lie on a continuum of increasingly less complex models. The most general model consists of pure ICA, in which none of the elements of **W** are constrained. When sources are modeled as Gaussian, **W** must produce Gaussians rows of **S** that are orthogonal to all other rows, which induces constraints on **W**. When Gaussian components are discarded in PCA, the criteria used for deciding the number of components to discard is typically based on finding a plateau in the amount of variance explained by successive components. This criterion can be formalized by modeling the discarded sources as having a single, uniform variance (effectively, white noise), thereby placing additional constraints on the number of freely adjustable parameters [40], [41], [48]. Recent neuroimaging work suggests that even estimating the correct Gaussian PCA signal remains a difficult and unresolved analytic problem in the presence of strong off-diagonal covariance terms found in brain networks [49]. At the extreme end of this continuum is a model with no non-Gaussian sources and uniform Gaussian variance such that **W** is replaced by a single parameter characterizing that variance. In principle, the ICA/PCA model could be extended to model a specified subset of Gaussian components as having uniform variance. Model selection could then be based on cross-validation estimates of T_*n*_, exactly as for ICA/PCA.

## Supporting Information

S1 Fig Sub-Gaussian source derived from the full craniometric data set. See Fig. 6 legend for formatting details.
(SVG)

S2 Fig Super-Gaussian source derived from the full craniometric data set. See Fig. 6 for formatting details.
(SVG)

S3 Fig Super-Gaussian source derived from the full craniometric data set. See Fig. 6 for formatting details.
(SVG)

S4 Fig Super-Gaussian source derived from the full craniometric data set. See Fig. 6 for formatting details.
(SVG)

S5 Fig Super-Gaussian source derived from the full craniometric data set. See Fig. 6 for formatting details.
(SVG)

S6 Fig Super-Gaussian source derived from the full craniometric data set. See Fig. 6 for formatting details.
(SVG)

S7 Fig Super-Gaussian source derived from the full craniometric data set. See Fig. 6 for formatting details.
(SVG)

S8 Fig Super-Gaussian source derived from the full craniometric data set. See Fig. 6 for formatting details.
(SVG)

S9 Fig Super-Gaussian source derived from the full craniometric data set. See Fig. 6 for formatting details.
(SVG)

S10 Fig Super-Gaussian source derived from the full craniometric data set. See Fig. 6 for formatting details.
(SVG)

S11 Fig Super-Gaussian source derived from the full craniometric data set. See Fig. 6 for formatting details.
(SVG)

S12 Fig Super-Gaussian source derived from the full craniometric data set. See Fig. 6 for formatting details.
(SVG)

S13 Fig Super-Gaussian source derived from the full craniometric data set. See Fig. 6 for formatting details.
(SVG)

S14 Fig Super-Gaussian source derived from the full craniometric data set. See Fig. 6 for formatting details.
(SVG)

S15 Fig Super-Gaussian source derived from the full craniometric data set. See Fig. 6 for formatting details.
(SVG)

S16 Fig Super-Gaussian source derived from the full craniometric data set. See Fig. 6 for formatting details.
(SVG)

S17 Fig Super-Gaussian source derived from the full craniometric data set. See Fig. 6 for formatting details.
(SVG)

S18 Fig Super-Gaussian source derived from the full craniometric data set. See Fig. 6 for formatting details.
(SVG)

S19 Fig Super-Gaussian source derived from the full craniometric data set. See Fig. 6 for formatting details.
(SVG)

S20 Fig Super-Gaussian source derived from the full craniometric data set. See Fig. 6 for formatting details.
(SVG)

S21 Fig Super-Gaussian source derived from the full craniometric data set. See Fig. 6 for formatting details.
(SVG)

S22 Fig Super-Gaussian source derived from the full craniometric data set. See Fig. 6 for formatting details.
(SVG)

S23 Fig Super-Gaussian source derived from the full craniometric data set. See Fig. 6 for formatting details.
(SVG)

S24 Fig Super-Gaussian source derived from the full craniometric data set. See Fig. 6 for formatting details.
(SVG)

S25 Fig Super-Gaussian source derived from the full craniometric data set. See Fig. 6 for formatting details.
(SVG)

S26 Fig Super-Gaussian source derived from the full craniometric data set. See Fig. 6 for formatting details.
(SVG)

S27 Fig Super-Gaussian source derived from the full craniometric data set. See Fig. 6 for formatting details.
(SVG)

S28 Fig Super-Gaussian source derived from the full craniometric data set. See Fig. 6 for formatting details.
(SVG)

S29 Fig Super-Gaussian source derived from the full craniometric data set. See Fig. 6 for formatting details.
(SVG)

S30 Fig Super-Gaussian source derived from the full craniometric data set. See Fig. 6 for formatting details.
(SVG)

S31 Fig Super-Gaussian source derived from the full craniometric data set. See Fig. 6 for formatting details.
(SVG)

S32 Fig Super-Gaussian source derived from the full craniometric data set. See Fig. 6 for formatting details.
(SVG)

S33 Fig Super-Gaussian source derived from the full craniometric data set. See Fig. 6 for formatting details.
(SVG)

S34 Fig Super-Gaussian source derived from the full craniometric data set. See Fig. 6 for formatting details.
(SVG)

S35 Fig Super-Gaussian source derived from the full craniometric data set. See Fig. 6 for formatting details.
(SVG)

S36 Fig Super-Gaussian source derived from the full craniometric data set. See Fig. 6 for formatting details.
(SVG)

S37 Fig Super-Gaussian source derived from the full craniometric data set. See Fig. 6 for formatting details.
(SVG)

S38 Fig Super-Gaussian source derived from the full craniometric data set. See Fig. 6 for formatting details.
(SVG)

S39 Fig Super-Gaussian source derived from the full craniometric data set. See Fig. 6 for formatting details.
(SVG)

S40 Fig Super-Gaussian source derived from the full craniometric data set. See Fig. 6 for formatting details.
(SVG)

S41 Fig Super-Gaussian source derived from the full craniometric data set. See Fig. 6 for formatting details.
(SVG)

S42 Fig Gaussian component from the full craniometric data set. See Fig. 6 for formatting details.
(SVG)

S43 Fig Gaussian component from the full craniometric data set. See Fig. 6 for formatting details.
(SVG)

S44 Fig Gaussian component from the full craniometric data set. See Fig. 6 for formatting details.
(SVG)

S45 Fig Gaussian component from the full craniometric data set. See Fig. 6 for formatting details.
(SVG)

S46 Fig Gaussian component from the full craniometric data set. See Fig. 6 for formatting details.
(SVG)

S47 Fig Gaussian component from the full craniometric data set. See Fig. 6 for formatting details.
(SVG)

S48 Fig Gaussian component from the full craniometric data set. See Fig. 6 for formatting details.
(SVG)

S49 Fig Gaussian component from the full craniometric data set. See Fig. 6 for formatting details.
(SVG)

S50 Fig Gaussian component from the full craniometric data set. See Fig. 6 for formatting details.
(SVG)

S51 Fig Gaussian component from the full craniometric data set. See Fig. 6 for formatting details.
(SVG)

S52 Fig Gaussian component from the full craniometric data set. See Fig. 6 for formatting details.
(SVG)

S53 Fig Gaussian component from the full craniometric data set. See Fig. 6 for formatting details.
(SVG)

S54 Fig Gaussian component from the full craniometric data set. See Fig. 6 for formatting details.
(SVG)

S55 Fig Gaussian component from the full craniometric data set. See Fig. 6 for formatting details.
(SVG)

S56 Fig Gaussian component from the full craniometric data set. See Fig. 6 for formatting details.
(SVG)

S57 Fig Gaussian component from the full craniometric data set. See Fig. 6 for formatting details.
(SVG)

S58 Fig Gaussian component from the full craniometric data set. See Fig. 6 for formatting details.
(SVG)

S59 Fig Gaussian component from the full craniometric data set. See Fig. 6 for formatting details.
(SVG)

S60 Fig Gaussian component from the full craniometric data set. See Fig. 6 for formatting details.
(SVG)

S61 Fig Gaussian component from the full craniometric data set. See Fig. 6 for formatting details.
(SVG)

S62 Fig Gaussian component from the full craniometric data set. See Fig. 6 for formatting details.
(SVG)

S63 Fig Gaussian component from the full craniometric data set. See Fig. 6 for formatting details.
(SVG)

S64 Fig Gaussian component from the full craniometric data set. See Fig. 6 for formatting details.
(SVG)

S65 Fig Gaussian component from the full craniometric data set. See Fig. 6 for formatting details.
(SVG)

S66 Fig Gaussian component from the full craniometric data set. See Fig. 6 for formatting details.
(SVG)

S67 Fig Gaussian component from the full craniometric data set. See Fig. 6 for formatting details.
(SVG)

S68 Fig Gaussian component from the full craniometric data set. See Fig. 6 for formatting details.
(SVG)

S69 Fig Gaussian component from the full craniometric data set. See Fig. 6 for formatting details.
(SVG)

S70 Fig Gaussian component from the full craniometric data set. See Fig. 6 for formatting details.
(SVG)

S71 Fig Gaussian component from the full craniometric data set. See Fig. 6 for formatting details.
(SVG)

S72 Fig Heat map relating z-scores to sources and components for the full craniometric data set. See Fig. 11 for formatting details. The first column corresponds to the Sub-Gaussian source shown in S1 Fig. Columns 2–41 correspond to the Super-Gaussian sources in the same order as shown in S2–S41 Figs. Columns 42–71 correspond to the Gaussian components in the same order as shown in S42–S71 Figs.
(TIF)

S1 Table Coefficients to compute reduced dimension non-Gaussian source scores and Gaussian component scores from the raw measures. (XLS)

S2 Table Coefficients to compute full dimension non-Gaussian source scores and Gaussian component scores from the raw measures. (XLS)
