# Supplementary figures and images for: How Many Separable Sources? Model Selection In Independent Components Analysis
Source: PLoS One. 2015 Mar 26;10(3):e0118877. doi: 10.1371/journal.pone.0118877 (PMC4374758; doi:10.1371/journal.pone.0118877)

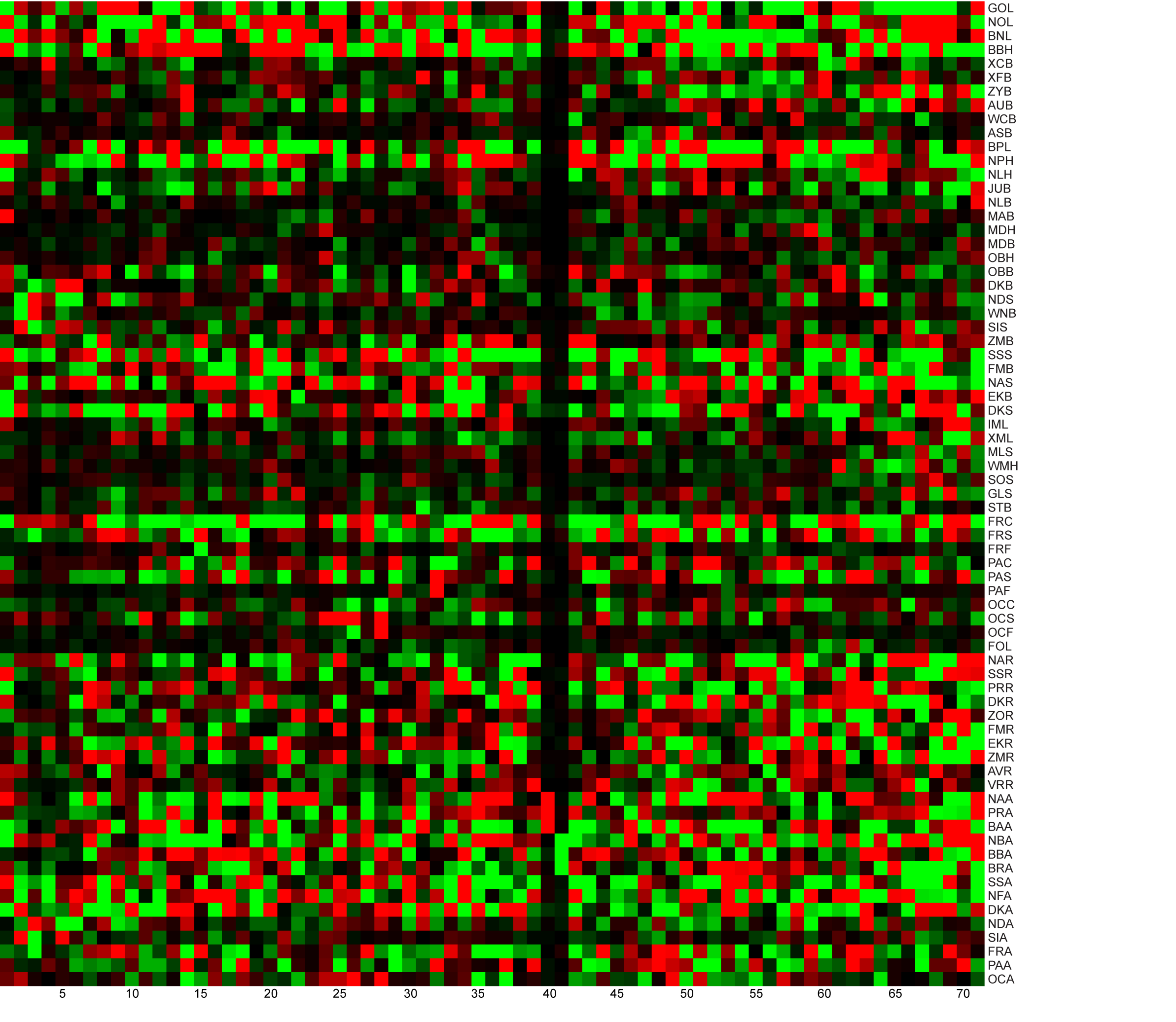

Supplement: S72 Fig — See Fig. 11 for formatting details. The first column corresponds to the Sub-Gaussian source shown in S1 Fig. Columns 2–41 correspond to the Super-Gaussian sources in the same order as shown in S2–S41 Figs. Columns 42–71 correspond to the Gaussian components in the same order as shown in S42–S71 Figs. (TIF) [file pone.0118877.s072.tif]
